# Investigation of a Medical Plant for Hepatic Diseases with Secoiridoids Using HPLC and FT-IR Spectroscopy for a Case of *Gentiana rigescens*

**DOI:** 10.3390/molecules25051219

**Published:** 2020-03-09

**Authors:** Yuangui Yang, Yanli Zhao, Zhitian Zuo, Ji Zhang, Yao Shi, Yuanzhong Wang

**Affiliations:** 1 Institute of Medicinal Plants, Yunnan Academy of Agricultural Sciences, Kunming 650200, China; ygyang2015@126.com (Y.Y.); yanli034120144@126.com (Y.Z.); zjyaas@hotmail.com (J.Z.); shiyyaas@163.com (Y.S.); 2 Institute of Chinese Materia Medica, Shanghai University of Traditional Chinese Medicine, Shanghai 201203, China

**Keywords:** hepatic diseases, secoiridoids, *G. rigenscens*, HPLC, FT-IR, PLSR

## Abstract

Secoiridoids could be used as a potential new drug for the treatment of hepatic disease. The content of secoiridoids of *G. rigescens* varied in different geographical origins and parts. In this study, a total of 783 samples collected from different parts of *G. rigescens* in Yunnan, Sichuan, and Guizhou Provinces. The content of secoiridoids including gentiopicroside, swertiamarin, and sweroside were determined by using HPLC and analyzed by one-way analysis of variance. Two selected variables including direct selected and variable importance in projection combined with partial least squares regression have been used to establish a method for the determination of secoiridoids using FT-IR spectroscopy. In addition, different pretreatments including multiplicative scatter correction (MSC), standard normal variate (SNV), first derivative and second derivative (SD), and orthogonal signal correction (OSC) were compared. The results indicated that the sample (root, stem, and leaf) with total secoiridoids, gentiopicroside, swertiamarin, and sweroside from west Yunnan had higher content than samples from the other regions. The sample from Baoshan had more total secoiridoids than other samples for the whole medicinal plant. The best performance using FT-IR for the total secoiridoid was with the direct selected variable method involving pretreatment of MSC+OSC+SD in the root and stem, while in leaf, of the best method involved using original data with MSC+OSC+SD. This method could be used to determine the bioactive compounds quickly for herbal medicines.

## 1. Introduction

Secoiridoids, which are natural monoterpenes compounds based on the cyclopentane, are made of iridoid in the cleavage of skeleton carbon chain with C_7_ and C_8_. They include gentiopicroside, swertiamarin, sweroside, and morroniside as part of Chinese herbal medicines which belong to *Gentiana* L (Gentianaceae family), a perennial herb [1]. Those compounds have been used for in hepatoprotective, anti-inflammatory, antipyretic, antiviral, and antioxidative treatments in the previous investigation [2,3]. Among them, hepatoprotective has been a vital part of research in recent years. As an important hepatoprotective therapeutic agent, secoiridoids could be used to inhibit liver damage. Gentiopicroside attenuated D-galactosamine/lipopolysaccharide induced apoptosis of hepatocytes and reduced in serum TNF-α [4]. Swertiamarin inhibited oxidative stress which leads to hepatic steatosis induced gluconeogenesis and lipogenesis [5]. Moreover, swertiamarin attenuates hepatic fibrosis by inhibiting hepatic stellate cells [6]. Gentiopicroside regulated the bile acids-related transporters to mitigate cholestasis [7]. It has similarly been reported that sweroside can increase the excretion of fecal bile acid [8]. Therefore, secoiridoids could be used as a potential new drug for the treatment of hepatic diseases.

Up to now, phytochemical research indicated that secoiridoids are mainly distributed in the plant of *G. rigescens* [9]. Therapeutic herbal medicine has had an effect on jaundice involving a damp-heat pathogen and inflammation of eyes for thousands of years [10]. The research has reported that secoiridoids of *G. rigescens* varied across different geographical origins and parts [11]. Despite this, detailed and deep investigations of secoridosids in *G. rigescens* are scarce. *G. rigescens*, also known as ‘Jian Long Dan’, is distributed in the southwestern regions of China. Various environmental conditions have a great influence on the chemical profiles [12]. So, it is necessary to investigate the secoridosids of *G. rigescens* comprehensively.

Various analytical methods including high performance thin-layer chromatography [13], high performance liquid chromatography (HPLC) [14], capillary electrophoresis [15], and liquid chromatography-mass spectrometry [16] have been used to determine the nature of secoridosides and its derivative. While some of these methods have complicated operations, their long analytical times remain useful and have been studied. Simple and comprehensive spectroscopic methods also have been used to measure secoridosides in *G. rigescens*. It is reported that gentiopicroside, swertiamarin, and sweroside quantities were determined using infrared spectroscopy coupled with partial least squares regression (PLSR) [17]. Wu et al. determined the gentiopicroside content of *G. rigescens*, demonstrating that Fourier transform infrared (FT-IR) spectroscopy and support vector machines could be applied to quantify bioactive components [18]. However, some irrelevant chemical information bad performances during the determination of bioactive compounds. Variable selection can not only improve the estimation accuracy but enhance the model’s interpretability [19]. Some selection methods including variable importance in projection (VIP) [20], a selectivity ratio [21], the successive projections algorithm [22], and the direct selected model [23] have been used to determinate compounds for samples. Among these methods, the direct selected method could improve the model profile for the measurement of total steroid saponins of *Paris*. Moreover, VIP could reduce the number of relevant variables needed for the detection of olive oil adulteration using FT-IR.

In this investigation, a total of 783 samples have been collected from different parts of *G. rigescens* in Yunnan, Sichuan, and Guizhou Provinces. The content of secoiridoids including gentiopicroside, swertiamarin, and sweroside were measured by HPLC. The amount of secoridosides was compared by using one-way analysis of variance with different parts and geographical origins. In addition, selected variables including the use of the directed selected method and VIP analysis have been utilized to establish a method for the determination of secoiridoids by using FT-IR.

## 2. Materials and Methods

### 2.1. Meterial and Reagents

**Figure 1:**
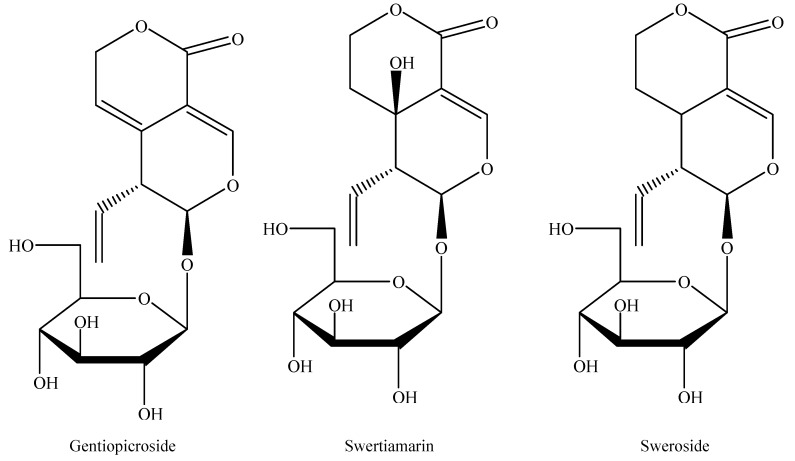
The structures of the reference standards.

The plant of *G. rigescens* is a herbaceous plant and is found under the pine tree and beside weeds at elevations of 1300-3000 m. Southwestern China (Yunnan, Guizhou, Sichuan Province) and its relative regions have been investigated. As shown in Table S1, there were 576 individuals in Yunnan Province (234, 50, 74, 91, 97, 30 distributed in the central, northeastern, northwestern, western, southeastern, and southwestern regions, respectively), along with 119 in Guizhou Province, 58 in Sichuan Province, 20 in Guangxi Province, and 10 in Hunan Province. Fresh materials were divided into three parts (root, stem, and leaf) and dried at room temperature. Sample powder was placed through 60 meshes and stored in a valve bag prior to further analysis. Reference standards (gentiopicroside, swertiamarin, and sweroside) were purchased from the Chinese National Institute for the Control of Pharmaceutical and Biological Products (Beijing, China). The purity determined by HPLC-MS was more than 98%. The structures of three standards are shown in Figure 1. Acetonitrile and formic acid (Farifield, CT) were HPLC grade solvent. Water was provided by Wahaha Co. Ltd. (Hangzhou, China). Potassium bromide in the spectroscopic grade was purchased by Tianjin Fengchuan Fine Chemical Research Institutes (Tianjin, China). Other chemical solvents were of analytical grade.

### 2.2. HPLC Analysis

A sample powder of 0.1 g was extracted with ultrasonication by using 2 mL of 80% methanol for 30 min. After standing for a few minutes, the lost methanol was added. Then the solvents were filtered by a 0.22 µm membrane filter. Each of the 5 µL was injected into an Agilent 1260 HPLC system (Agilent Technologies, Santa Clara, California, USA) equipped with a G1315D diode-array detector. The separation HPLC method was altered based on a method outline in a published article [14]. Chromatographic separation was carried out using a Shimadzu Intersil-C_18_ column (150 × 4.6 mm, 5 µm). The mobile phase used acetonitrile (A) and 0.1% formic acid in water (B). The flow rate was set at 1 mL/min. The gradient strategy was set as follows: 0–7% A, for the first 0.4 min; 7%–10% A, for 0.4–2.5 min; 10%–26% A, for 2.5–20 min; 26%–58.2%A, for 20–29.02 min; 58.2%–90% A, for 29.02–30 min, and 90%A, for 30–34 min. Column temperature was maintained at 30 °C and the detective wavelength was set at 246 nm.

### 2.3. FT-IR Spectroscopy

Each sample of 1.5±0.02 mg was mixed with potassium bromide (100 mg) in the agate mortar. FT-IR spectra were collected by Perkin-Elmer FT-IR Spectrometer (Norwald, CT, USA) equipped with deuterated triglycine sulfate detector. Before the analysis, the instrument was preheated 30 min under 65% relative humidity. Each spectrum was scanned in the absorbance in a range from 4000 to 400 cm^−1^.

In order to obtain good model performance, different pretreatment methods including multiplicative scatter correction (MSC), standard normal variate (SNV), first derivative (FD), second derivative (SD), and orthogonal signal correction (OSC) were investigated. SNV was used to remove the interferences of scatter and particle size [24]. As an alternative approach, MSC could also reduce particle size effect [25]. FD and SD were used to eliminate baseline drifts and enhance small spectral signals [26]. OSC could remove un-related signal and improve the predictivity and interpretability of the solution [27].

### 2.4. Method Validation

Standard references (gentiopicroside of 3.6 mg, swertiamarin of 0.7 mg, sweroside of 0.7 mg) was extracted using 1 mL of methanol. The solution was diluted with methanol into different six concentrations and injected into the HPLC-DAD system in triplicate. Plotting the peak area (y) against the concentrations of composition (x) developed a standard calibration curve for the determination of content of each compound in the samples. The limit of detection (LOD) and limit of quantification (LOQ) were equal to a signal-to-noise of 3 and 10, respectively. For inter-day precision, sample was analyzed six times (0, 2, 4, 8, 12. 24 h) each day over three consecutive days for intra-day precision. Recovery was analyzed using stock solutions spiked with low, medium, and high concentrations. It was calculated by using Equation (1):

Recovery rate = (measured amount-added amount)/original amount × 100%.(1)

### 2.5. PLSR

#### 2.5.1. Data Partition of Calibration and Validation Set

In this study, a total of 154 samples for different regions and parts were used. Based on the SPXY algorithm, those samples were divided into a calibration set (103 samples) and a validation set (51 samples). As shown in Table 1, the lowest and highest content of secoiridoid for the original date of root in the calibration set were 0.72 and 88.68 mg/g, and 0.97 and 73.68 mg/g for the validation set. This indicated that the content of secoiridoid in the calibration covered that in the validation set. Similar results could be found in the other data set.

#### 2.5.2. Variable Selected

**Figure 2:**
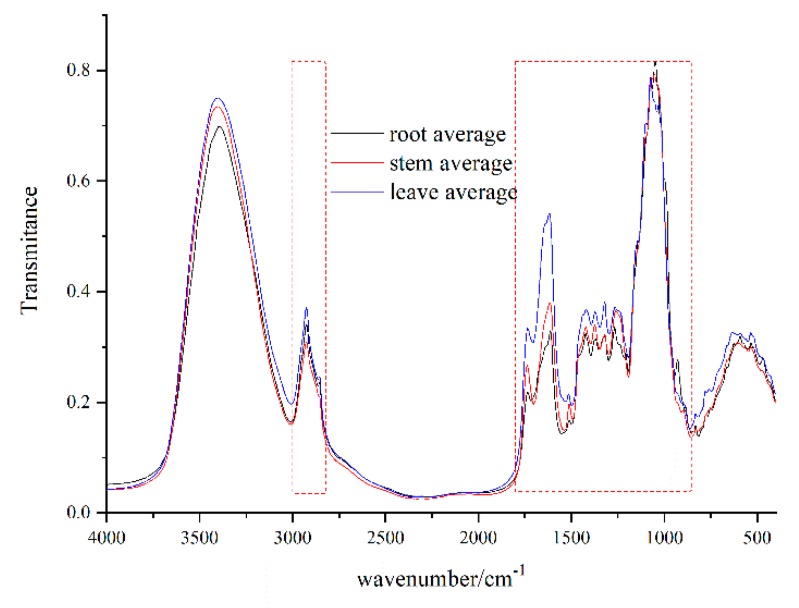
The average spectrum of root, stem, and leaf for *G. rigescens* from different geographical areas.

**Figure 3:**
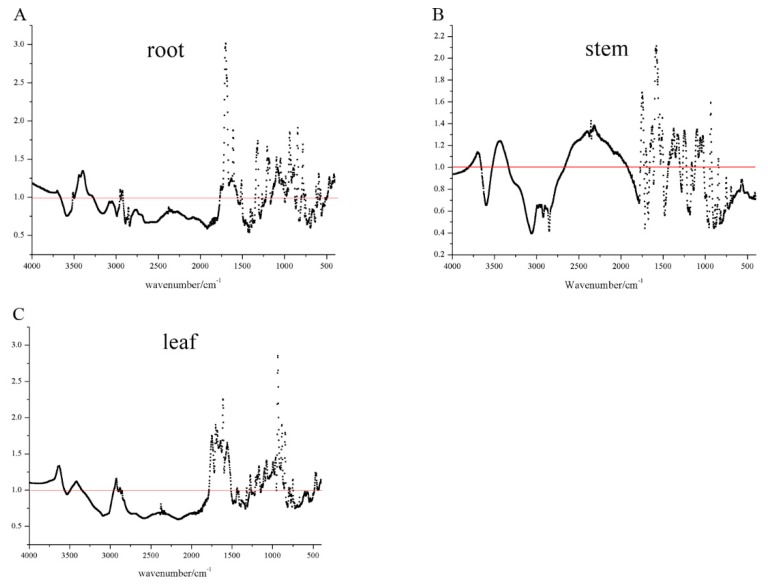
VIP variable screening for root (**A**), stem (**B**), and leaf (**C**) (VIP of more than 1 was selected).

To have a better performance, two variable selected methods including direct selected analysis and VIP analysis were investigated in this study. For the direct selected method, wavenumbers in the ranges of 820-1780 cm^−1^ and 2810-3010 cm^−1^ (Figure 2) was discussed. Nuclear parents of peaks were assigned as the following: 2930 cm^−1^ (-CH_2_ asymmetric stretching); 2848 cm^−1^ (-CH_2_ stretching); 1611 cm^−1^ (C=C stretching); 1457 cm^−1^ (-CH_2_ bending); 1268 cm^−1^ (C-O stretching); and 1075 cm^−1^ (-C-OH stretching). Among them, absorption peaks at 1075 and 1611 cm^−1^ are characteristic of iridoids skeletal vibration according to a previous study [28]. For VIP analysis, the average of the squared VIP scores equals 1, while a value more than 1 is considered as the criterion for variable selection [29]. In this study, a matrix based on different pretreatment spectra was subjected to VIP variables screening. As shown in Figure 3A–C, values over 1 in the root, stem, leaf spectroscopy was selected. Then, the selected variables were used to develop a new database for further study.

#### 2.5.3. Model Evaluation

To evaluate the performance of the model, some parameters including root mean square error of calibration (RMSEC), root mean square error of prediction (RMSEP), residual predictive deviation (RPD), determination coefficients of calibration (R_c_^2^), and determination coefficients of prediction (R_p_^2^) were calculated. RMSEC and RMSEP could express the prediction error in the internal and external validation [30]. The lower their value was, the better their performance in the calibration and validation. RMSE could be calculated as Equation (2). R_c_^2^ and R_p_^2^ responded to the prediction ability of the model [31]. Good performance for the model occurred when the values of R_c_^2^ and R_p_^2^ were similar to 1. R^2^ is equal to the value obtained through Equation (3). RPD was used to test the precision and accuracy of the model. A high RPD value was considered to indicate a robust model. The higher value of RPD was, the greater the prediction of the model. When the value of RPD was more than 2, it indicated that the model was good for calibration [32]. RPD could be calculated by using Equation (4).

(2) $$\mathrm{RMSE}=\sqrt{\frac{\sum_{i=1}^{n}{\left(x_{i}{-y}_{i}\right)}^{2}}{n}}$$

(3) $$R^{2}=\frac{\sum_{i=1}^{n}{\left(x_{i}{-y}_{i}\right)}^{2}}{\sum_{i=1}^{n}{\left(x_{i}-x\right)}^{2}}$$

(4) $$\mathrm{RPD}=\frac{\mathrm{STDEV}}{\mathrm{RMSEP}}$$

where n is the number of samples in the calibration or validation set, x_i_ is the measured result for the calibration or validation set of sample i, and y_i_ is the predicted result for the calibration or validation set of sample i. STDEV is the standard deviation.

### 2.6. Data Analysis

HPLC data analysis was conducted by the Fingerprint of Traditional Chinese Medicine (Version 2004A), Chinese Pharmacopoeia Committee. To compare the content of secoridosides in the different geographical areas and parts, ANOVA and a Tukey’s test at *P* < 0.05 were carried out by the R 3.4.0 program (R Core Team, 2017). Different pretreatments of FT-IR data including MSC, SNV, FD, SD, OSC, as well as the PLSR model were calculated by SIMCA-P^+^ (Version 13.0, Umetrics, Sweden). RMSEC, RMSEP, R_c_^2^, R_p_^2^, and RPD were calculated by using Microsoft Excel 2013 (Redmond, WA).

## 3. Results and Discussion

### 3.1. Optimization of HPLC Conditions

**Figure 4:**
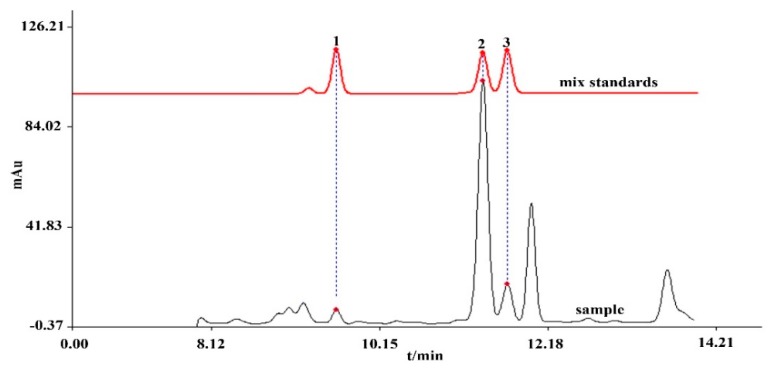
HPLC chromatography of *G. rigescens*. Peak 1 (swertiamarin), peak 2 (gentiopicroside), and peak 3 (sweroside) for mixed standards and samples.

The Shimadzu Inertsil ^®^ ODS-C_18_ column (4.6×150 mm, 5 µm) had better performance in the separation of secoiridoids of *G. rigescens* Waters XBridge ^®^ ODS-C_18_ column (4.6 × 150 mm, 5 µm). The column temperature in the range of 25 to 35 °C was optimized, and the results indicated that a temperature 30 °C had a higher resolution than others. The high flow rate could have a good resolution, while it could lead to high pressure in the HPLC system. The final flow rate was set at 1 mL/min. In addition, the wavelengths investigated were at 232 nm, 241 nm, 243 nm, 246 nm, 258 nm 275 nm, and 300 nm. The results indicated that secoiridoids had adequate absorption at 246 nm. The typical chromatography of *G. rigensces* is shown in Figure 4.

### 3.2. Method Validation

As shown in Table 2, the inter- and intra-day precision of relative common peaks were below 2.92% and 2.74%. The recovery rate was calculated in the range of 96%–101.08% with a RSD less than 2.68% shown in Table 3. This indicated that the method for quantitative analysis of secoiridoids had precise and accurate results.

### 3.3. Secoiridoids Origin from Southwestern China

**Figure 5:**
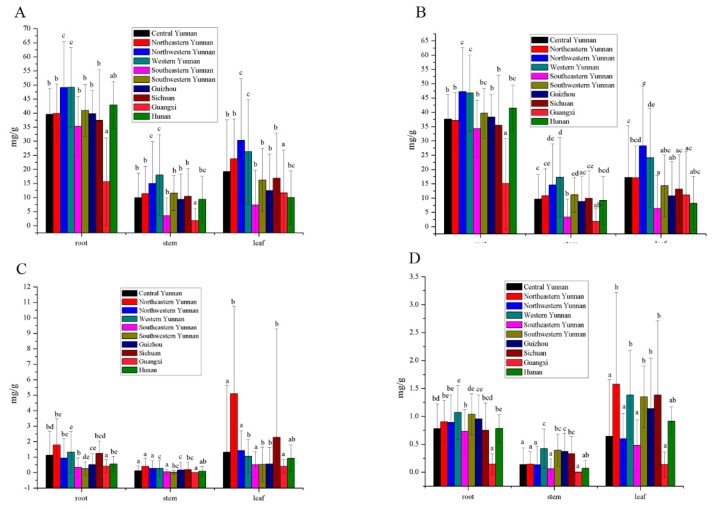
Different parts (root, stem, and leaf) in the content of total secoiridoids (**A**), gentiopicroside (**B**), sweroside (**C**), and swertiamarin (**D**) of *G. rigescens* in southwestern China (*P* < 0.05).

The content of gentiopicroside, sweroside, and swertiamarin in 783 samples from different geographical origins and parts are shown in Table S2. Different parts (the root, stem, and leaf) in the content of total secoiridoids of *G. rigescens* in southwestern China varied significantly. As shown in Figure 5A, the highest content of secoiridoids was in the root, and the lowest was in the stem. Various amounts of secoiridoids were discovered in the same part of the southwestern region of China. Secoiridoids associated with roots in Guangxi Province were lower than in other regions (*P* < 0.05). Samples from central, northeastern, southeastern, and southwestern Yunnan, Guizhou, Sichuan, and Hunan had the similar content levels (*P* > 0.05). In addition, the content levels in the northwestern and western Yunnan Province were the highest (*P* < 0.05). The varied content of secoiridoids in the stem and leaf from the different regions had the same trend. This indicated that geographical origin has a great influence on the content of secoiridoids. The content of gentiopicroside in the different regions and parts are shown in Figure 5B. The trend was as similar to that of secoiridoids in root. The lowest and highest contents of gentiopicroside were from Guangxi, as well as northwestern and western Yunnan Province (*P* < 0.05). These areas had different amounts of gentiopicroside from those of other areas. The content in the stem was different in different regions. Samples from Guangxi had lower contents than in other areas except for Guizhou (*P* < 0.05). The content in western Yunnan was 9.67 times higher than that in Guangxi (*P* < 0.05). For the leaf, gentiopicroside had the highest content in northwestern Yunnan, which was 4.46 times higher than for the southeastern region (*P* < 0.05). The order of content for gentiopicroside were root, leaf, and stem. The results were not in accordance with a previous study which showed that the highest content is in leaves of a regenerated plantlet; the reason for this may be that leaves have materials with a long growing time, thereby accumulating active compound sufficiently over time [16]. In addition, the average content of gentiopicroside in previous study could reach up to 122.93 mg/g, which below the 47.23 mg/g found in this investigation. The reason for this is that the large number of samples may respond to the actual level. For the sweroside (Figure 5C), its content varied slightly across different parts and regions. While leaf samples from northeastern Yunnan were abnormal, this could not explain the phenomenon found in this study. Interestingly, it was found that the compound contents from southeastern Yunnan and Guizhou were lowest (*P* < 0.05), while the highest levels were found in western Yunnan. The content of swertiamarin was too low to detect in some samples (Figure 5D). Samples (root, stem and leaf) from western Yunnan had a higher content than in other regions (*P* < 0.05). The same results could be found for total secoiridoids, gentiopicroside, and sweroside. It was therefore necessary for samples from western Yunnan Province to be investigated next.

### 3.4. Secoiridoids Origin from Northwestern and Western Yunnan Province

**Figure 6:**
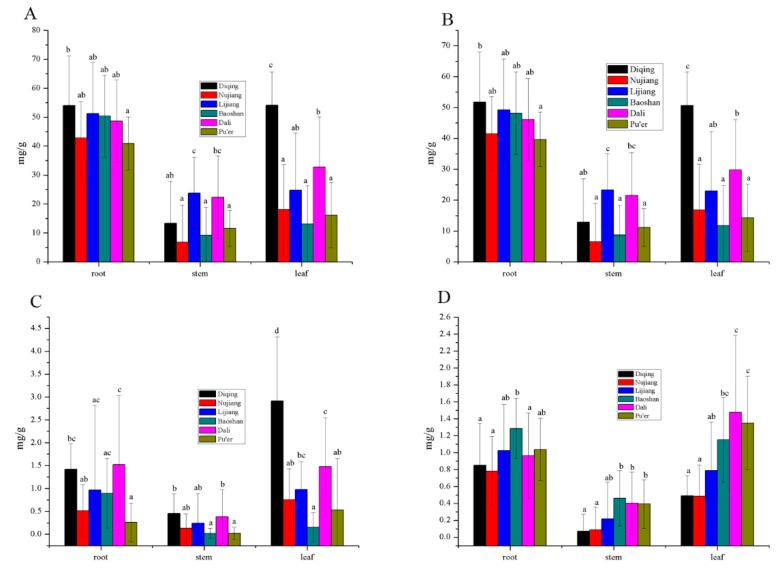
Different parts (root, stem, and leaf) in the content of total secoiridoids (**A**), gentiopicroside (**B**), sweroside (**C**), and swertiamarin (**D**) of *G. rigescens* in the western Yunnan Province (*P* < 0.05).

Northwestern and western Yunnan Province including Diqing, Nujiang, Lijiang, Baoshan, Dali, and Pu’er have varying conditions in their terrains and elevations. To obtain the actual results, a total of 197 samples were investigated. As shown in Figure 6A, the content of total secoiridoids changed significantly in the root, while changing slightly in the stem and leaf. The content in the root was higher than that in other parts. For the total secoiridoids, the content of total secoridoids from Diqing with a root was higher than that from Pu’er (*P* < 0.05), and other regions had the same content. The compound of stems from Lijiang and Dali were greater than that from others. In the leaf, the number of total secoridoids from Diqing was highest among western Yunnan Province (*P* < 0.05). As a main compound, gentiopicroside had the same trend to total secoridoids (Figure 6B). As shown in Figure 6C, the content of sweroside was below 1.5 mg/g in different regions, except for leaves in Diqing. Some samples were too low to detect, such as stems from Nujiang, Baoshan, and Pu’er, as well as leaves from Baoshan. For the swertiamarin (Figure 6D), the number of samples from Baoshan was higher than for other regions (*P* < 0.05) involving roots. Moreover, in the stem and leaf, the content from Baoshan, Dali, and Pu’er was higher than for Diqing and Nujiang (*P* < 0.05). This indicated that samples from Baoshan had more total secoiridoids than other samples for the whole medicinal plant.

### 3.5. PLSR for Total Secoiridoids

**Figure 7:**
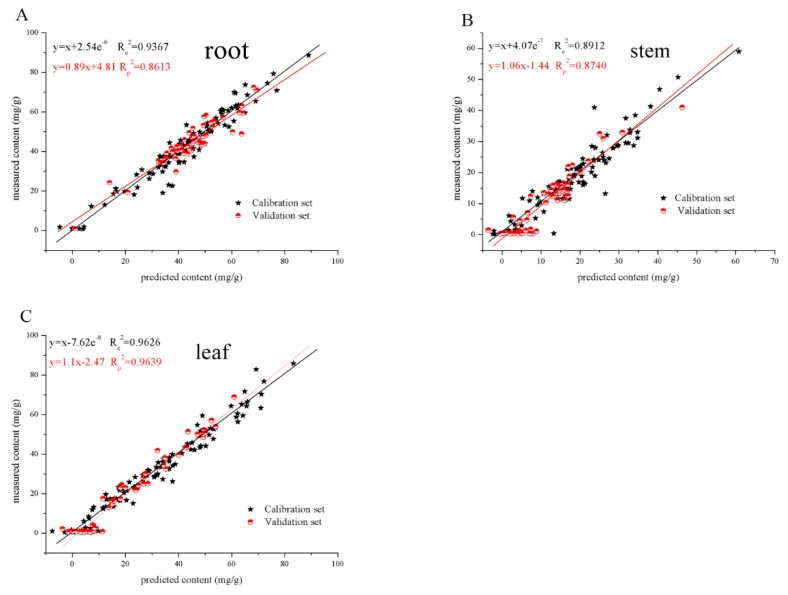
The relationship between predicted and measured content for total secoiridoids in the root (**A**), stem (**B**) and leaf (**C**).

To develop a fast determination method for total secoiridoids, various variables were selected and pretreatments combined with the PLSR model have been used. As shown in Table 4, five pretreatments including SNV, MSC, MSC+OSC, MSC+OSC+FD, and MSC+OSC+SD were compared. For different pretreatments, MSC and SNV could improve the value of R_c_^2^, R_p_^2^, and RPD, and decrease RMSEE. While the value of RPD in the most of model was lower than 2, it had the worst performance when predicting the total quantity of secoridoids. However, the data was subjected by MSC+OSC, the RPD value was higher than 2, indicating a good performance. The results were in accordance with a previous study which showed that OSC could remove the irrelative information for the prediction model [23]. When the data was handled by the combination of pretreatments MSC+OSC+FD and MSC+OSC+SD, it had better performance than others in terms of RMSEE, RMSEP, R_c_^2^, R_p_^2^, and RPD. This indicated that combined processing could have synergetic effects on models for classification and regression [33]. Three different variables including the origin, direct selected, and VIP analysis methods were compared for predictions of total secoiridoids of different parts. The selected variable method could improve the model performance when compared with original data in the root. VIP analysis could decrease the number of variables, while it could not have effects on the model. The best performance for the total secoiridoids was for a selected variable with pretreatment of MSC+OSC+SD, which had RMSEE, RMSEP, R_c_^2^, R_p_^2^, and RPD of 4.5757, 4.5731, 0.9367, 0.8613, and 2.6298, respectively (Figure 7A). The same trend could be found for total secoiridoids involving stems, and the best performance was selected involving a variable with MSC+OSC+SD. Parameters RMSEE, RMSEP, R_c_^2^, R_p_^2^, and RPD were 3.9018, 3.5715, 0.8912, 0.874, and 2.5886, respectively (Figure 7B). However, for the total secoiridoids of leaves, the selection method had no effect on the model of prediction. The best performance showed original data with MSC+OSC+SD, parameters RMSEE, RMSEP, R_c_^2^, R_p_^2^, and RPD were 4.2230, 4.2420, 0.9626, 0.9639, and 4.5041, respectively (Figure 7C).

## 4. Conclusions

Secoiridoids have been used as a potential new drug for the treatment of hepatic diseases. A total of 783 samples collected from different parts of *G. rigescens* in Yunnan, Sichuan, and Guizhou were investigated using HPLC and FT-IR spectroscopy. Various selected variables including direct selected and VIP analysis methods have been used to establish a method for the determination of secoiridoids for FT-IR spectroscopy. The results indicated that the highest content of secoiridoids was in roots, and the lowest was in stems. In the PLSR for total secoiridoids, it could be found that the best performance for the total secoiridoids of roots used selected variables with pretreatment of MSC+OSC+SD, which had RMSEE, RMSEP, R_c_^2^, R_p_^2^, and RPD of 4.5757, 4.5731, 0.9367, 0.8613, and 2.6298, respectively.

## Figures and Tables

**Table 1:** The summary of secoiridoids in the calibration and validation sets.

		Min (mg/g)	Max (mg/g)	Mean (mg/g)	SD
root-origin	Calibration set	0.72	88.68	43.35	18.02
	Validation set	0.97	73.68	43.76	11.55
root-selected	Calibration set	0.72	88.68	42.84	18.00
	Validation set	0.97	72.43	44.73	11.82
root-VIP	Calibration set	0.72	88.68	43.36	17.98
	Validation set	0.97	73.68	43.72	11.84
stem-origin	Calibration set	0.29	58.96	18.04	11.82
	Validation set	0.29	33.78	11.25	9.61
stem-selected	Calibration set	0.29	58.96	17.96	11.77
	Validation set	0.30	41.01	11.41	9.84
stem-VIP	Calibration set	0.29	58.96	17.21	12.03
	Validation set	0.29	41.01	12.92	10.05
leaf-origin	Calibration set	0.34	85.88	30.95	21.63
	Validation set	0.57	68.89	21.56	20.28
leaf-selected	Calibration set	0.34	85.88	31.55	22.58
	Validation set	0.41	53.33	20.37	17.37
leaf-VIP	Calibration set	0.34	85.88	32.78	21.70
	Validation set	0.43	53.33	17.90	17.72

**Table 2:** The regression equation, linearity range, correlation coefficient (r^2^), LOD, LOQ, inter-day, and intra-day standards.

Analyte	Regression Equation	Linearity Range (μg/mL)	r^2^	LOD (μg/mL)	LOQ (μg/mL)	Inter-Day	Intra-Day
gentiopicroside	Y = 5826.56946x + 130.94542	24.42–3600.00	0.9998	52.58	175.25	1.54	1.89
swertiamarin	Y = 8161.85182x + 11.73637	1.79–184.47	0.9995	1.45	4.83	2.32	1.62
sweroside	Y = 4129.62105x + 2.48267	1.75–343.00	0.9987	3	9.98	2.92	2.74

**Table 3:** The recovery rate of gentiopicroside, swertiamarin, and sweroside.

	Original Amount (mg/g)	Added Amount (mg/g)	Measured Amount (mg/g)	Recovery Rate	RSD
		0.5	1.71	96%	2.33%
gentiopicroside	1.23	1	2.21	98%	2.68%
		1.5	2.72	99.33%	2.42%
		24	72.78	101.08%	1.90%
swertiamarin	48.52	48	95.76	98.42%	0.85%
		60	107.74	98.70%	1.68%
		1	2.88	99%	1.19%
sweroside	1.89	1.5	3.34	96.67%	2.88%
		3	4.83	98%	1.80%

**Table 4:** Various variable selected and pretreatments combined with PLSR for total secoiridoids.

		LV	RMSEE	RMSEP	R_c_^2^	R_p_^2^	RPD
root-origin	original	8	8.5139	6.2829	0.7964	0.7038	1.7020
	SNV	7	7.8257	5.8600	0.8243	0.7472	1.8820
	MSC	7	7.7996	5.7331	0.8254	0.7544	1.9240
	MSC+OSC	1	5.3947	4.3041	0.9112	0.8592	2.5492
	MSC+OSC+1st	2	4.3754	4.8940	0.9422	0.8443	2.4392
	MSC+OSC+2st	2	4.4507	4.7914	0.9402	0.8546	2.4626
root-selected	original	6	8.5225	7.7012	0.7893	0.6156	1.5250
	SNV	5	8.2827	7.6118	0.7968	0.6508	1.5844
	MSC	5	8.1629	7.6997	0.8107	0.5557	1.3806
	MSC+OSC	2	5.2748	4.6584	0.9159	0.8635	2.5795
	MSC+OSC+1st	2	4.5626	4.6172	0.9379	0.8497	2.5132
	**MSC+OSC+2st**	**2**	**4.5757**	**4.5731**	**0.9367**	**0.8613**	**2.6298**
root-VIP	original	7	8.7822	6.5822	0.7779	0.6893	1.6299
	SNV	5	8.3730	6.0865	0.7938	0.7406	1.8118
	MSC	5	8.2735	5.8589	0.7967	0.7576	1.8793
	MSC+OSC	2	5.7787	5.4884	0.8987	0.7898	2.0616
	MSC+OSC+1st	2	5.3411	5.1036	0.9135	0.8162	2.2285
	MSC+OSC+2st	2	5.4466	5.0488	0.9100	0.8174	2.2791
stem-origin	original	3	10.0467	9.7660	0.2985	0.1919	0.8185
	SNV	7	7.2516	7.8903	0.6496	0.5301	1.1515
	MSC	7	7.2809	7.9455	0.6466	0.5364	1.1513
	MSC+OSC	2	5.2292	5.3134	0.8103	0.7316	1.7194
	MSC+OSC+1st	3	3.9330	3.9500	0.8925	0.8445	2.3729
	MSC+OSC+2st	2	3.0003	3.6079	0.9368	0.8779	2.4998
stem-selected	original	6	7.8168	9.4903	0.5851	0.2504	0.9290
	SNV	4	7.5040	8.2579	0.6096	0.3795	1.0452
	MSC	4	7.4939	8.2921	0.6149	0.3684	1.0386
	MSC+OSC	2	5.7072	4.6995	0.7695	0.7855	1.9185
	MSC+OSC+1st	2	4.8656	4.4198	0.8325	0.8159	2.1008
	**MSC+OSC+2st**	**2**	**3.9018**	**3.5715**	**0.8912**	**0.8740**	**2.5886**
stem-VIP	original	5	8.7443	8.9892	0.4925	0.3219	0.8713
	SNV	2	10.0687	8.6813	0.3130	0.3039	0.9061
	MSC	4	8.6653	8.4545	0.5006	0.3693	1.0021
	MSC+OSC	2	5.4675	4.7228	0.7974	0.7830	1.9927
	MSC+OSC+1st	1	5.3223	4.3811	0.8061	0.8187	2.1707
	MSC+OSC+2st	1	5.2983	4.7727	0.8079	0.7815	1.9946
leaf-origin	original	8	10.3486	9.9320	0.7892	0.7839	1.8854
	SNV	6	10.5555	8.7191	0.7777	0.8236	2.1817
	MSC	6	10.3409	9.0157	0.7850	0.8221	2.1101
	MSC+OSC	3	5.9238	6.0009	0.9272	0.9144	3.3800
	MSC+OSC+1st	3	5.3764	4.5992	0.9400	0.9529	4.4631
	**MSC+OSC+2st**	**2**	**4.2230**	**4.2420**	**0.9626**	**0.9639**	**4.5041**
leaf-selected	original	6	10.1826	9.9332	0.8086	0.7360	1.5651
	SNV	3	10.7515	10.4739	0.7799	0.7053	1.5485
	MSC	3	10.7445	10.5360	0.7802	0.7058	1.5375
	MSC+OSC	1	6.6718	6.8725	0.9135	0.8600	2.5923
	MSC+OSC+1st	2	5.0529	4.7375	0.9509	0.9248	3.6239
	MSC+OSC+2st	1	5.3409	4.9566	0.9446	0.9294	3.5038
leaf-VIP	original	9	8.9449	11.9643	0.8453	0.6701	1.3841
	SNV	6	8.7314	9.9045	0.8478	0.7799	1.6555
	MSC	6	8.7607	9.8223	0.8467	0.7856	1.6692
	MSC+OSC	2	6.4200	6.0348	0.9142	0.9082	2.9345
	MSC+OSC+1st	2	5.9801	6.4896	0.9250	0.8860	2.7377
	MSC+OSC+2st	2	5.4105	6.3621	0.9391	0.8872	2.7910

## Supplementary Materials

The following are available online, Table S1: Different geographical origin of *G. rigescens.* Table S2: The content of swertiamarin, gentiopicroside, sweroside of *Gentiana rigescens* in the different geographical origin and parts (mg/g).
