# Supplementary material for: Investigation of a Medical Plant for Hepatic Diseases with Secoiridoids Using HPLC and FT-IR Spectroscopy for a Case of Gentiana rigescens
Source: Molecules. 2020 Mar 9;25(5):1219. doi: 10.3390/molecules25051219 (PMC7179471; doi:10.3390/molecules25051219)
Supplement: Supplementary file 1 [file molecules-25-01219-s001.pdf]

Investigation a medical plant for hepatic diseases with secoiridoids using HPLC and FT-IR spectroscopy -as a case of *Gentiana rigescens*

Yuangui Yang[1,2], Yanli zhao[1], Zhitian Zuo[1*], Ji Zhang[1], Yao Shi[1] and Yuanzhong Wang[1*]

[1] Institute of Medicinal Plants, Yunnan Academy of Agricultural Sciences, Kunming 650200, China
[2] Institute of Chinese Materia Medica, Shanghai University of Traditional Chinese Medicine, Shanghai 201203, China

Table S1. Different geographical origin of *G. rigescens*.

	Cite	Number	Latitude and Longitude		Altitude	Collected time
Central Yunnan	Xishan District of Kunming	10	N25°3′22.1″	E102°32′41.2″	2150	2012.11.10
	Yiliang Country of Kunming	10	N25°02′57.7″	E103°17′39.6″	1817	2012.12.28
	Shilin Country of Kunming	10	N24°42′47.6″	E103°36′19.5″	2144	2012.12.28
	Guandu District of Kunming	10	N25°19′62.57″	E102°52′38.01″	2292	2013.1.7
	Jinning Country of Kunming	9	N24°31′21.98″	E102°31′02.80″	2287	2013.1.8
	Wuhua District of Kunming	9	N25°11′15.4″	E102°39′49.1″	2023	2013.1.16
	Fumin Country of Kunming	10	N25°23′24.3″	E102°26′47.0″	2205	2013.1.16
	Songming Country of Kunming	9	N25°20′37.2″	E102°52′11.4″	2248	2013.1.23
	Xinping Country of Yuxi	10	N23°58′01.1″	E101°56′57.1″	2016	2012.12.18
	Chengjiang Country of Yuxi	10	N24°44′53.0″	E102°53′22.8	2643	2013.1.9
	Yuanjiang Country of Yuxi	8	N23°40′00.6″	E101°46′13.7″	2150	2013.1.9
	E'shan Country of Yuxi	9	N24°08′19.4″	E102°15′26.1″	1945	2013.1.16
	Yimen Country of Yuxi	9	N24°51′58.40″	E102°07′30.63″	1876	2013.1.12
	Dayao Country of Chuxiong	10	N25°40′06.3″	E101°36′44.4″	1804	2013.1.16
	Nanhua Country of Chuxiong	10	N25°05′05.4″	E101°16′51.9″	1969	2013.1.16
	Lufeng Country of Chuxiong	7	N25°01′57.4″	E102°10′25.9″	2082	2013.1.16
	Nanhua Country of Chuxiong	9	N25°18′39.93″	E101°16′15.40″	2105	2013.1.12
	Yao'an Country of Chuxiong	10	N25°38′32.70″	E101°06′21.24″	2370	2013.1.13
	Yao'an Country of Chuxiong	9	N25°23′24.39″	E101°16′42.48″	2220	2013.1.12
	Luoping Country of Qujing	10	N25°11′43.0″	E104°9′59.3″	2270	2012.12.1
	Zhanyi Country of Qujing	9	N25°40′21.72″	E103°40′2.94″	2038	2012.12.19

	Shizong Country of Qujing	10	N24°38′17.6″	E104°09′06.9″	2256	2012.12.28
	Fuyuan Country of Qujing	10	N25°25′04.7″	E104°10′27.5″	1954	2012.12.28
	Longma Country of Qujing	11	N25°27′19.12″	E103°24′41.83″	1938	2013.1.23
	Xuanwei City of Qujing	6	N26°00′18.66″	E104°09′06.53″	2063	2013.1.25
Northeastern Yunnan	Xundian Country of Kunming	10	N25°32′17.53″	E103°19′56.34″	1906	2012.12.20
	Luquan Country of Kunming	11	N25°55′04.67″	E102°51′55.30″	2241	2013.1.7
	Dongchuan District of Kunming	11	N25°56′39.55″	E103°03′53.75″	2592	2013.1.7
	Wuding Country of Kunming	9	N25°30′51.6″	E102°07′46.2″	2072	2013.1.16
	Qiaojia Country of Zhaotong	9	N27°03′28.2″	E102°58′28.0″	2531	2013.1.23
Northwestern Yunnan	Weixi Country of Diqing	10	N27°19′45.4″	E99°16′41.2″	2893	2012.12.6
	Weixi Country of Diqing	10	N27°31′05.5″	E99°22′10.3″	2520	2012.12.6
	Lanping Country of Nujiang	10	N26°34′36.6″	E99°26′32.5″	2978	2012.12.18
	Lanping Country of Nujiang	6	N26°32′37.6″	E99°27′33.5″	2610	2012.12.18
	Lushui Country of Nujiang	10	N25°51′14.1″	E99°51′20.4″	2852	2012.12.18
	Yulong Country of Lijiang	12	N27°20′25.2″	E100°59′01.1″	2256	2012.12.6
	Ninglang Country of Lijiang	10	N27°20′25.2″	E100°59′01.1″	3219	2012.12.6
	Gucheng District of Lijiang	3	N26°40′33.65″	E100°15′59.77″	2390	2013.1.30
	Gucheng District of Lijiang	3	N26°46′01.92″	E100°16′42.36″	2416	2013.1.30
Western Yunnan	Tengchong Country of Baoshan	10	N24°58′56.9″	E98°29′18.6″	1963	2012.12.18
	Longling Country of Baoshan	10	N24°48′39.5″	E99°47′10.2″	2821	2012.12.18
	Changning Country of Baoshan	10	N24°54′20.8″	E99°22′53.0″	1770	2012.12.18
	Binchuan Country of Dali	11	N25°56′39.5″	E100°22′15.3″	2460	2012.11.24
	Dali Cangshan Global Geopark	10	N25°39′03.9″	E100°09′48.9″	2225	2012.11.24
	Er'hai Country of Dali	10	N25°59′44.2″	E99°54′46.5″	2350	2012.11.24
	Heqing Country of Dali	10	N26°29′49.8″	E100°17′00.3″	2505	2012.11.24
	Yongping Country of Dali	10	N25°29′33.7″	E99°38′17.5″	2356	2012.12.18
	Nanjian Country of Dali	10	N24°57′01.1″	E100°28′10.7″	1914	2012.12.18
Southeastern Yunnan	Luxi Country of Honghe	10	N24°24′51.4″	E103°41′16.3″	1891	2013.1.9
	Mile Country of Honghe	10	N24°15′16.1″	E103°37′26.0″	2015	2013.1.9
	Mengzi Country of Honghe	10	N23°24′27.4″	E103°43′50.2″	1987	2013.1.9
	Pingbian Country of Honghe	10	N23°15′38.40″	E103°41′26.11″	1796	2013.1.9
	Gejiu City of Honghe	10	N23°46′43.2″	E102°48′49.4″	1945	2013.1.9

	Honghe Country of Honghe	9	N23°14′14.12″	E102°23′49.02″	1971	2013.1.9
	Qiubei Country of Wenshan	9	N24°01′39.44″	E104°11′52.88″	1879	2013.1.9
	Yanshan Country of Wenshan	10	N23°42′47.2″	E104°14′54.9″	1615	2013.1.9
	Wenshan City of Wenshan	10	N23°18′58.7″	E104°08′32.3″	1788	2013.1.9
Southwestern	Jingdong Country of Pu'er	10	N24°29′40.2″	E100°47′05.8″	2520	2012.12.18
Yunnan	Zhenyuan Country of Pu'er	10	N23°55′25.5″	E101°06′14.3″	2311	2012.12.18
	Mojiang Country of Pu'er	10	N23°56′45.1″	E101°13′36.1″	1704	2012.12.18
Guizhou	Qishe Town of Xingyi	10	N25°09′41.1″	E104°50′24.3″	2158	2012.12.28
	Baiwanyao Town of Xingyi	11	N25°04′36.5″	E104°46′22.3″	2028	2012.12.28
	Sala Town of Bijie	10	N27°14′33.7″	E105°07′41.8″	1933	2013.10.10
	Dafang Country of Bijie	9	N27°11′51.3″	E105°42′23.0″	1663	2013.10.10
	Qianxi Country of Bijie	9	N26°56′00.0″	E105°55′54.0″	1454	2013.10.10
	Zhongshan District of Anshun	10	N26°38′29.4″	E104°42′37.5″	2473	2013.10.10
	Kaiyang Country of Guiyang	10	N25°53′33.1″	E105°28′56.2″	1562	2013.10.24
	Zhanjie Town of Qingzhen	10	N27°01′35.2″	E106°19′09.0″	1600	2013.10.24
	Longli Country of Douyun	10	N26°38′22.6″	E104°43′26.0″	1590	2013.10.24
	Longli Country of Douyun	10	N26°33′33.6″	E106°54′59.2″	1393	2013.11.5
	Longli Country of Qiannan	10	N26°27′38.7″	E106°55′40.8″	1300	2013.11.5
	Taijiang Country of Qiannan	10	N26°33′36.2″	E108°19′45.5″	1260	2013.11.5
Sichuan	Dajing Town of Xichang	8	N27°42′36.8″	E102°21′30.4″	2258	2013.1.30
	Zhaojue Country of Xichang	10	N27°51′03.5″	E102°25′50.3″	2601	2013.1.30
	Xide Country of Liangshan	10	N28°23′58.50″	E102°25′2.93″	2716	2013.1.30
	Mianning Country of Liangshan	10	N28°31′27.2″	E102°12′19.3″	1921	2013.1.30
	Yanbian Country of Panzhihua	10	N27°09′35.0″	E101°16′26.5″	2807	2013.1.30
	Hanyuan Country of Ya'an	10	N29°21′17.29″	E102°54′1.56″	1916	2013.11.6
Guangxi	Longlin Country of Baise	10	N24°36′56″	E105°05′20″	1571	2013.11.28
	Napo Country of Baise	10	N23°23′16.44″	E105°50′57.70″	1110	2013.11.6
Hunan	Bucheng Country of Shaoyang	10	N26°09′26″	E110°07′38″	1788	2013.11.28

Table S2 The content of swertiamarin, gentiopicroside, sweroside of *Gentiana rigescens* in the different geographical origin and parts (mg/g).

		Root			Stem			Leaf		
		swertiamarin	gentiopicroside	sweroside	swertiamarin	gentiopicroside	sweroside	swertiamarin	gentiopicroside	sweroside
Central Yunnan	Xishan District of Kunming	0.00	33.13	0.76	0.00	0.00	0.00	0.00	58.66	0.00
	Xishan District of Kunming	0.71	43.20	1.15	0.00	24.22	0.00	0.00	52.35	0.00
	Xishan District of Kunming	0.33	39.82	0.98	0.00	23.72	0.00	0.00	39.57	0.00
	Xishan District of Kunming	0.44	41.41	1.20	0.00	0.00	0.00	0.00	43.05	0.00
	Xishan District of Kunming	0.46	38.55	0.76	0.00	29.53	0.00	0.00	70.27	0.00
	Xishan District of Kunming	0.00	24.17	0.00	0.00	20.13	0.00	0.00	85.88	0.00
	Xishan District of Kunming	0.00	23.06	0.00	0.00	37.55	0.00	0.00	76.80	0.00
	Xishan District of Kunming	0.00	32.20	0.00	0.00	11.55	0.00	0.00	56.36	0.00
	Xishan District of Kunming	0.00	27.52	0.73	0.00	17.99	0.00	0.00	63.34	0.00
	Xishan District of Kunming	0.39	32.32	0.00	0.00	0.00	0.00	0.00	93.90	0.00
	Yiliang Country of Kunming	1.01	46.10	0.87	0.00	13.95	0.00	0.34	30.20	1.25
	Yiliang Country of Kunming	0.64	29.71	0.75	0.00	24.94	1.00	0.53	38.49	1.79
	Yiliang Country of Kunming	1.56	53.60	2.42	0.00	20.29	1.02	0.37	12.85	2.36
	Yiliang Country of Kunming	0.88	49.69	0.61	0.00	12.76	0.00	0.30	23.95	1.24
	Yiliang Country of Kunming	0.56	41.93	0.00	0.00	22.96	0.00	0.38	14.12	0.99
	Yiliang Country of Kunming	0.88	49.12	0.75	0.00	22.14	0.00	0.43	16.67	1.53
	Yiliang Country of Kunming	0.00	35.91	0.00	0.00	18.15	0.00	0.00	15.25	1.13
	Yiliang Country of Kunming	0.00	35.14	0.73	0.00	16.10	0.00	0.00	19.34	1.27
	Yiliang Country of Kunming	0.95	47.75	1.53	0.00	20.94	0.00	0.52	28.15	1.61
	Yiliang Country of Kunming	0.96	50.58	1.08	0.00	13.98	0.00	0.00	40.21	0.00
	Shilin Country of Kunming	0.00	34.45	0.74	0.00	17.14	0.00	0.41	55.73	1.58
	Shilin Country of Kunming	0.96	42.62	1.46	0.00	20.47	0.00	0.00	31.64	1.05
	Shilin Country of Kunming	0.00	31.99	0.65	0.00	11.28	0.72	0.00	24.52	1.75

Shilin Country of Kunming	0.00	33.59	1.82	0.00	12.43	0.00	0.32	31.75	1.04
Shilin Country of Kunming	0.32	32.27	0.75	0.00	0.00	0.00	0.00	17.09	1.34
Shilin Country of Kunming	0.88	40.05	1.63	0.00	10.55	0.00	1.02	12.25	1.16
Shilin Country of Kunming	0.46	35.56	1.70	0.00	18.96	0.61	0.45	17.61	1.32
Shilin Country of Kunming	0.56	31.34	1.41	0.00	14.82	0.00	0.89	13.57	0.90
Shilin Country of Kunming	0.73	32.10	1.43	0.00	10.57	0.69	0.00	0.00	1.45
Shilin Country of Kunming	0.61	31.10	1.40	0.00	0.00	0.69	0.00	0.00	1.49
Guandu District of Kunming	0.94	44.33	0.78	0.00	10.61	0.00	0.00	28.11	0.69
Guandu District of Kunming	0.89	37.66	0.93	0.00	0.00	0.00	0.00	29.07	0.93
Guandu District of Kunming	0.67	31.33	0.75	0.00	20.11	0.00	0.56	28.30	0.65
Guandu District of Kunming	0.68	33.21	0.67	0.00	10.66	0.00	0.42	19.65	0.76
Guandu District of Kunming	0.52	36.68	0.77	0.00	10.94	0.00	0.00	0.00	0.00
Guandu District of Kunming	0.66	39.01	1.12	0.00	16.18	0.00	0.00	0.00	1.03
Guandu District of Kunming	0.47	43.56	1.04	0.00	12.41	0.00	0.00	0.00	0.00
Guandu District of Kunming	0.60	43.44	1.21	0.00	13.51	0.00	0.00	30.31	1.41
Guandu District of Kunming	0.50	31.68	0.00	0.00	13.68	0.00	0.31	17.03	0.99
Guandu District of Kunming	0.64	27.75	1.01	0.00	0.00	0.00	0.00	12.94	0.84
Jinning Country of Kunming	0.94	41.84	0.00	0.00	0.00	0.00	0.69	0.00	0.00
Jinning Country of Kunming	0.76	37.67	0.00	0.00	0.00	0.00	0.71	0.00	0.00
Jinning Country of Kunming	1.06	44.95	0.00	0.91	19.05	0.00	0.50	0.00	0.00
Jinning Country of Kunming	1.37	48.42	0.00	0.65	0.00	0.64	0.43	0.00	0.00
Jinning Country of Kunming	0.95	43.10	0.67	0.00	0.00	0.00	0.96	0.00	0.00
Jinning Country of Kunming	1.60	44.50	0.74	0.00	0.00	0.00	0.00	0.00	0.00
Jinning Country of Kunming	1.61	57.58	1.56	0.40	11.94	0.00	0.65	0.00	0.00
Jinning Country of Kunming	1.20	41.21	0.00	0.67	18.85	0.00	0.72	0.00	0.00
Jinning Country of Kunming	1.67	56.78	0.76	0.00	0.00	0.00	0.50	0.00	0.00
Wuhua District of Kunming	0.00	25.56	0.00	0.00	19.66	0.00	0.00	17.40	0.00

Wuhua District of Kunming	0.40	36.45	1.20	0.00	14.18	0.00	0.00	13.92	1.15
Wuhua District of Kunming	0.38	29.63	0.00	0.00	12.27	0.00	0.29	18.43	0.66
Wuhua District of Kunming	0.46	42.06	0.97	0.00	21.50	0.00	0.00	0.00	0.00
Wuhua District of Kunming	0.49	37.41	0.00	0.00	15.73	0.00	0.00	11.27	0.00
Wuhua District of Kunming	0.54	36.46	0.00	0.00	15.43	0.00	0.00	0.00	0.00
Wuhua District of Kunming	0.30	25.98	0.00	0.00	14.71	0.00	0.00	0.00	0.63
Wuhua District of Kunming	0.35	30.23	0.00	0.00	20.18	0.00	0.00	16.23	0.62
Wuhua District of Kunming	0.45	32.79	0.76	0.00	20.20	0.00	0.00	0.00	0.00
Fumin Country of Kunming	0.79	40.41	0.83	0.00	0.00	0.00	0.00	21.43	0.80
Fumin Country of Kunming	0.88	41.84	0.67	0.00	0.00	0.00	0.30	0.00	0.00
Fumin Country of Kunming	0.87	42.34	1.06	0.00	0.00	0.00	0.52	17.80	1.14
Fumin Country of Kunming	0.69	29.31	0.62	0.00	11.96	0.00	0.00	29.41	0.97
Fumin Country of Kunming	0.48	30.61	0.92	0.00	0.00	0.00	0.00	0.00	0.62
Fumin Country of Kunming	0.79	50.41	2.00	0.00	21.36	0.00	0.00	20.35	0.67
Fumin Country of Kunming	1.47	40.29	1.55	0.00	0.00	0.00	0.00	38.22	0.00
Fumin Country of Kunming	1.60	47.38	2.54	0.00	11.32	0.00	0.00	34.13	0.00
Fumin Country of Kunming	0.68	35.99	2.15	0.00	0.00	0.00	0.60	61.41	2.21
Fumin Country of Kunming	0.65	39.27	1.33	0.00	0.00	0.00	0.00	24.07	0.84
Songming Country of Kunming	0.38	45.16	1.30	0.00	14.03	0.00	0.00	17.88	0.88
Songming Country of Kunming	1.10	41.59	1.23	0.00	12.61	0.00	0.00	35.80	0.91
Songming Country of Kunming	1.08	45.63	1.13	0.00	17.95	0.00	0.00	19.82	1.18
Songming Country of Kunming	0.74	33.31	1.36	0.00	18.77	0.00	0.00	13.16	1.09
Songming Country of Kunming	1.40	41.91	0.84	0.00	0.00	0.00	0.68	34.56	1.34
Songming Country of Kunming	0.74	31.52	0.70	0.00	11.46	0.00	0.00	13.12	0.74
Songming Country of Kunming	0.56	39.41	0.78	0.00	13.06	0.00	0.00	12.17	0.97
Songming Country of Kunming	0.69	38.80	0.90	0.00	19.67	0.00	0.00	14.66	0.00
Songming Country of Kunming	0.55	47.38	1.58	0.00	0.00	0.00	0.00	27.81	0.76

Xinping Country of Yuxi	1.22	43.88	0.76	0.00	13.93	0.00	0.44	28.77	0.00
Xinping Country of Yuxi	1.10	50.68	0.92	0.00	14.52	0.00	0.86	35.21	0.00
Xinping Country of Yuxi	1.33	44.29	0.84	0.00	0.00	0.00	0.96	23.52	0.00
Xinping Country of Yuxi	1.23	40.90	1.13	0.30	0.00	0.00	0.66	0.00	0.00
Xinping Country of Yuxi	0.90	35.52	0.00	0.49	13.81	0.00	1.86	23.13	0.00
Xinping Country of Yuxi	1.02	39.15	0.65	0.46	27.38	0.00	0.83	33.42	0.00
Xinping Country of Yuxi	1.00	46.87	0.64	0.00	10.95	0.00	0.00	0.00	0.00
Xinping Country of Yuxi	1.23	41.38	1.19	0.41	23.43	0.61	1.70	21.65	1.72
Xinping Country of Yuxi	1.16	40.47	0.73	0.69	20.66	0.00	2.15	34.07	2.08
Xinping Country of Yuxi	0.96	36.96	0.77	0.00	11.06	0.00	1.22	0.00	0.00
Chengjiang Country of Yuxi	1.13	39.33	0.74	0.00	0.00	0.00	1.42	10.99	3.73
Chengjiang Country of Yuxi	1.36	46.04	0.95	0.00	17.74	0.00	0.42	0.00	9.89
Chengjiang Country of Yuxi	1.28	35.59	0.00	0.00	0.00	0.00	0.68	10.70	13.06
Chengjiang Country of Yuxi	0.00	27.11	0.00	0.00	0.00	0.00	1.69	0.00	1.11
Chengjiang Country of Yuxi	1.04	36.15	1.43	0.00	14.68	0.00	1.37	0.00	2.69
Chengjiang Country of Yuxi	1.30	46.37	1.47	0.32	0.00	0.00	1.87	12.02	3.45
Chengjiang Country of Yuxi	1.26	47.02	0.67	0.00	15.97	0.00	1.19	0.00	7.40
Chengjiang Country of Yuxi	1.21	40.84	0.93	0.00	0.00	0.00	2.11	0.00	3.11
Chengjiang Country of Yuxi	1.27	50.78	0.66	0.45	13.56	0.00	1.43	0.00	3.03
Chengjiang Country of Yuxi	1.06	43.98	0.78	0.51	19.51	0.00	1.73	0.00	3.82
Yuanjiang Country of Yuxi	0.37	36.22	0.00	0.00	0.00	0.00	0.00	16.59	0.71
Yuanjiang Country of Yuxi	0.86	39.86	0.00	0.00	0.00	0.00	1.14	16.90	0.00
Yuanjiang Country of Yuxi	0.82	38.07	0.00	0.00	15.27	0.00	1.04	19.83	0.00
Yuanjiang Country of Yuxi	1.57	61.08	0.60	0.00	0.00	0.00	0.40	0.00	0.00
Yuanjiang Country of Yuxi	1.09	46.70	0.00	0.00	0.00	0.00	0.66	0.00	0.00
Yuanjiang Country of Yuxi	1.12	46.07	0.90	0.00	0.00	0.00	0.31	0.00	0.00
Yuanjiang Country of Yuxi	0.93	40.34	0.00	0.00	13.28	0.00	0.73	15.99	0.00

Yuanjiang Country of Yuxi	0.50	34.99	0.00	0.00	0.00	0.00	0.42	22.88	0.00
E'shan Country of Yuxi	0.43	27.69	0.00	0.00	0.00	0.00	0.00	37.86	0.00
E'shan Country of Yuxi	0.00	25.08	0.00	0.00	0.00	0.00	0.00	19.45	0.00
E'shan Country of Yuxi	0.77	31.23	0.00	0.00	13.14	0.00	0.00	45.54	0.00
E'shan Country of Yuxi	0.00	18.53	0.00	0.00	0.00	0.00	0.32	21.43	0.00
E'shan Country of Yuxi	0.64	31.44	0.00	0.00	13.72	0.00	0.31	16.69	0.00
E'shan Country of Yuxi	0.48	31.49	0.75	0.00	0.00	0.00	0.00	33.32	1.39
E'shan Country of Yuxi	0.51	31.94	0.00	0.00	0.00	0.00	0.00	19.51	0.00
E'shan Country of Yuxi	0.44	27.28	0.00	0.00	15.91	0.00	0.00	12.48	0.99
E'shan Country of Yuxi	0.35	34.76	0.00	0.00	10.91	0.00	0.00	0.00	0.00
Yimen Country of Yuxi	0.71	34.71	0.00	0.00	11.47	0.00	0.00	0.00	0.00
Yimen Country of Yuxi	0.44	25.20	0.00	0.00	10.95	0.00	0.00	0.00	0.00
Yimen Country of Yuxi	0.48	31.98	0.73	0.00	0.00	0.00	0.00	0.00	0.67
Yimen Country of Yuxi	0.50	24.42	0.00	0.00	0.00	0.00	0.00	0.00	0.65
Yimen Country of Yuxi	0.33	27.98	1.12	0.00	0.00	0.00	0.00	0.00	0.95
Yimen Country of Yuxi	0.00	19.59	0.00	0.00	0.00	0.00	0.00	0.00	1.45
Yimen Country of Yuxi	1.14	39.93	1.86	0.00	0.00	0.00	0.00	0.00	0.00
Yimen Country of Yuxi	0.76	33.75	1.09	0.00	0.00	0.00	0.00	0.00	1.47
Yimen Country of Yuxi	0.00	24.39	0.00	0.00	0.00	0.00	0.00	0.00	0.00
Dayao Country of Chuxiong	0.82	29.91	3.23	0.00	0.00	0.00	0.33	0.00	0.00
Dayao Country of Chuxiong	0.78	35.09	4.21	0.00	0.00	0.00	0.00	0.00	0.77
Dayao Country of Chuxiong	0.96	39.35	6.60	0.00	15.51	1.03	0.00	0.00	0.99
Dayao Country of Chuxiong	1.00	37.81	4.64	0.00	0.00	0.00	0.00	0.00	1.07
Dayao Country of Chuxiong	0.72	38.70	2.40	0.00	11.65	0.00	0.00	0.00	1.04
Dayao Country of Chuxiong	0.76	44.15	7.60	0.00	0.00	0.00	0.00	0.00	0.85
Dayao Country of Chuxiong	0.88	32.36	8.43	0.00	0.00	0.00	0.00	0.00	1.01
Dayao Country of Chuxiong	0.79	41.70	6.42	0.00	15.51	0.86	0.35	0.00	1.09

Dayao Country of Chuxiong	0.72	32.51	2.22	0.00	0.00	0.00	0.45	0.00	1.09
Dayao Country of Chuxiong	0.59	31.28	3.29	0.00	0.00	0.00	0.35	0.00	1.07
Nanhua Country of Chuxiong	1.12	32.99	2.31	0.77	0.00	0.00	2.42	11.45	0.00
Nanhua Country of Chuxiong	1.85	45.76	2.03	1.63	20.36	1.30	2.03	17.24	0.71
Nanhua Country of Chuxiong	1.40	49.72	3.76	1.15	24.28	0.66	2.97	27.97	0.93
Nanhua Country of Chuxiong	1.17	42.24	0.99	0.56	0.00	0.00	2.81	33.53	0.86
Nanhua Country of Chuxiong	1.58	47.09	0.76	1.02	22.46	0.00	1.45	22.98	0.67
Nanhua Country of Chuxiong	1.59	43.26	0.72	0.52	0.00	0.00	1.49	20.31	0.00
Nanhua Country of Chuxiong	1.34	49.45	1.24	0.79	11.15	0.00	2.06	0.00	0.00
Nanhua Country of Chuxiong	1.19	41.55	0.79	0.99	17.85	0.00	2.71	41.57	0.66
Nanhua Country of Chuxiong	1.19	40.48	1.10	1.03	14.18	0.00	3.37	52.59	0.97
Nanhua Country of Chuxiong	1.40	43.71	1.78	0.65	15.33	0.00	2.33	25.87	0.74
Lufeng Country of Chuxiong	0.00	27.43	0.00	0.00	16.42	0.00	0.00	0.00	0.00
Lufeng Country of Chuxiong	0.37	29.54	0.00	0.00	0.00	0.00	0.00	0.00	0.00
Lufeng Country of Chuxiong	0.42	34.40	0.00	0.00	0.00	0.00	0.00	24.74	1.35
Lufeng Country of Chuxiong	0.00	36.67	0.00	0.00	0.00	0.00	0.00	0.00	0.00
Lufeng Country of Chuxiong	0.00	36.87	0.62	0.00	0.00	0.00	0.00	13.07	0.00
Lufeng Country of Chuxiong	0.00	36.28	0.72	0.00	13.01	0.00	0.00	25.98	0.68
Lufeng Country of Chuxiong	0.00	32.34	0.00	0.94	12.02	0.00	0.00	71.91	3.30
Nanhua Country of Chuxiong	0.93	49.74	2.65	0.00	0.00	0.00	0.55	0.00	0.70
Nanhua Country of Chuxiong	1.17	39.37	1.88	0.00	0.00	0.00	1.72	0.00	0.80
Nanhua Country of Chuxiong	1.50	40.95	11.01	0.00	10.95	1.24	1.10	14.33	7.55
Nanhua Country of Chuxiong	0.80	23.18	1.56	0.00	11.87	0.00	0.62	0.00	0.00
Nanhua Country of Chuxiong	0.59	35.41	1.07	0.00	0.00	0.00	0.00	0.00	0.65
Nanhua Country of Chuxiong	1.63	48.96	1.74	0.48	18.81	0.00	1.47	0.00	0.90
Nanhua Country of Chuxiong	0.39	29.36	2.19	0.00	0.00	0.82	0.42	0.00	1.08
Nanhua Country of Chuxiong	0.79	35.62	7.85	0.00	0.00	1.01	0.42	0.00	0.72

Nanhua Country of Chuxiong	0.47	39.45	4.44	0.00	11.02	0.78	0.00	0.00	1.00
Yao'an Country of Chuxiong	1.35	36.11	1.35	0.00	0.00	0.00	1.15	0.00	1.31
Yao'an Country of Chuxiong	1.08	24.57	1.35	0.34	17.95	1.16	2.37	0.00	4.39
Yao'an Country of Chuxiong	0.85	22.59	1.84	0.00	0.00	0.00	0.73	0.00	1.57
Yao'an Country of Chuxiong	1.11	27.46	2.09	0.00	0.00	1.16	0.50	10.91	55.64
Yao'an Country of Chuxiong	1.21	32.68	1.27	0.00	0.00	0.00	8.05	0.00	1.17
Yao'an Country of Chuxiong	0.80	23.38	2.58	0.00	0.00	0.00	0.30	0.00	1.04
Yao'an Country of Chuxiong	1.11	34.13	1.18	0.00	0.00	1.29	0.52	0.00	31.39
Yao'an Country of Chuxiong	1.49	45.08	3.46	0.00	0.00	0.00	0.87	0.00	1.58
Yao'an Country of Chuxiong	0.77	25.29	1.87	0.00	0.00	0.00	0.62	0.00	1.41
Yao'an Country of Chuxiong	0.53	26.10	1.13	0.00	11.48	0.00	0.61	0.00	1.82
Yao'an Country of Chuxiong	1.21	35.15	2.18	0.80	12.15	0.00	1.17	0.00	0.00
Yao'an Country of Chuxiong	1.03	27.56	0.77	0.36	0.00	0.00	6.06	24.02	2.64
Yao'an Country of Chuxiong	0.80	35.09	1.61	0.32	0.00	0.00	2.17	0.00	1.14
Yao'an Country of Chuxiong	0.79	27.56	0.66	0.53	12.81	0.00	1.60	20.39	2.88
Yao'an Country of Chuxiong	0.82	28.86	0.84	0.80	16.85	0.00	1.50	0.00	1.73
Yao'an Country of Chuxiong	1.04	35.61	3.27	0.77	13.74	1.44	1.45	18.71	2.71
Yao'an Country of Chuxiong	1.14	37.32	2.12	1.02	21.08	0.95	6.61	20.34	5.15
Yao'an Country of Chuxiong	0.70	28.22	1.93	0.00	0.00	0.00	0.00	0.00	0.00
Yao'an Country of Chuxiong	0.86	36.96	0.74	0.00	0.00	0.00	1.36	15.29	1.06
Luoping Country of Qujing	1.73	53.25	0.97	0.83	12.78	0.00	1.27	18.41	0.68
Luoping Country of Qujing	1.16	50.96	1.17	0.00	0.00	0.00	1.69	32.17	0.00
Luoping Country of Qujing	1.19	43.02	1.07	1.27	0.00	0.00	1.79	21.18	0.00
Luoping Country of Qujing	1.69	57.85	2.49	1.15	26.50	0.96	2.26	38.20	0.00
Luoping Country of Qujing	1.27	50.33	0.78	0.92	12.32	0.00	2.48	27.89	1.50
Luoping Country of Qujing	2.19	57.48	9.82	0.96	10.64	0.77	1.89	29.43	2.15
Luoping Country of Qujing	1.39	58.42	0.80	0.39	13.45	0.64	1.57	36.64	1.65

Luoping Country of Qujing	0.93	39.57	0.00	0.36	13.14	0.00	1.07	0.00	0.00
Luoping Country of Qujing	1.27	53.85	0.00	0.70	12.44	0.00	1.81	40.02	0.00
Luoping Country of Qujing	1.46	54.40	1.44	0.52	0.00	0.91	1.12	15.20	0.78
Zhanyi Country of Qujing	0.90	44.97	0.00	0.00	16.51	0.00	0.70	30.47	0.93
Zhanyi Country of Qujing	0.74	43.13	0.00	0.00	14.86	0.00	0.00	16.89	0.84
Zhanyi Country of Qujing	0.00	35.16	0.00	0.00	10.61	0.00	0.00	23.59	0.98
Zhanyi Country of Qujing	0.31	38.97	0.00	0.00	0.00	0.00	0.46	40.27	1.30
Zhanyi Country of Qujing	1.03	44.17	0.92	0.00	21.21	0.00	1.16	42.74	1.24
Zhanyi Country of Qujing	0.59	42.09	1.09	0.00	23.57	0.00	1.49	34.42	1.16
Zhanyi Country of Qujing	0.76	47.75	0.67	0.00	11.88	0.00	0.82	47.02	1.29
Zhanyi Country of Qujing	0.77	42.27	1.27	0.00	14.29	0.00	0.84	21.82	1.00
Zhanyi Country of Qujing	0.00	20.91	0.00	0.00	11.60	0.00	0.65	33.17	0.00
Shizong Country of Qujing	1.00	40.48	0.00	0.00	16.02	0.00	0.47	23.47	0.80
Shizong Country of Qujing	0.61	30.93	0.00	0.63	24.90	0.00	0.00	0.00	0.00
Shizong Country of Qujing	0.66	37.37	0.81	0.00	23.28	0.00	0.00	17.18	0.84
Shizong Country of Qujing	0.31	22.84	0.00	0.00	14.72	0.00	0.00	10.68	0.00
Shizong Country of Qujing	0.30	36.36	0.00	0.00	16.76	0.00	0.00	24.14	0.99
Shizong Country of Qujing	0.53	31.63	0.00	0.00	16.44	0.00	0.00	29.10	1.85
Shizong Country of Qujing	0.34	30.87	0.00	0.00	0.00	0.00	0.00	12.01	0.00
Shizong Country of Qujing	0.70	35.45	0.00	0.00	11.80	0.00	0.00	20.57	0.66
Shizong Country of Qujing	0.95	34.13	0.00	0.00	12.24	0.00	0.00	16.88	0.73
Shizong Country of Qujing	0.35	25.67	0.66	0.00	14.09	0.00	0.00	13.15	0.00
Fuyuan Country of Qujing	0.83	39.62	2.00	0.00	15.95	0.00	1.16	42.49	1.35
Fuyuan Country of Qujing	0.62	37.44	0.71	0.00	13.91	0.00	0.37	48.37	1.45
Fuyuan Country of Qujing	0.87	37.06	0.83	0.38	12.51	1.34	1.32	26.64	0.64
Fuyuan Country of Qujing	1.08	44.44	0.00	0.36	20.82	0.00	1.72	32.35	0.00
Fuyuan Country of Qujing	0.89	41.94	0.00	0.49	12.27	1.68	1.96	26.64	0.00

	Fuyuan Country of Qujing	0.96	52.00	1.39	0.00	13.61	0.00	0.78	30.29	1.29
	Fuyuan Country of Qujing	0.00	38.30	0.68	0.00	12.42	0.00	0.33	28.84	0.74
	Fuyuan Country of Qujing	1.02	44.50	0.00	0.00	18.66	0.00	0.62	45.67	1.16
	Fuyuan Country of Qujing	1.04	40.50	0.00	0.00	13.61	0.00	0.70	25.64	0.00
	Fuyuan Country of Qujing	0.00	24.34	0.00	0.00	12.53	0.00	0.41	27.60	0.85
	Longma Country of Qujing	0.67	36.83	1.04	0.00	12.20	0.00	0.00	0.00	0.00
	Longma Country of Qujing	0.00	0.00	0.00	0.61	25.64	0.73	0.46	24.12	0.73
	Longma Country of Qujing	1.45	49.11	1.89	0.00	0.00	0.00	0.33	0.00	0.00
	Longma Country of Qujing	0.60	26.66	0.72	0.00	0.00	0.00	0.00	13.86	0.00
	Longma Country of Qujing	0.54	31.33	1.05	0.00	17.07	0.00	0.36	22.60	1.27
	Longma Country of Qujing	0.45	31.70	0.84	0.00	18.47		0.76	0.00	0.66
	Longma Country of Qujing	0.66	35.29	1.04	0.00	11.38	0.00	0.00	25.51	0.82
	Longma Country of Qujing	0.69	28.77	1.47	0.00	11.37	0.00	0.89	32.61	1.82
	Longma Country of Qujing	0.85	33.93	1.12	0.00	0.00	0.00	0.49	26.95	1.43
	Longma Country of Qujing	0.97	35.39	1.21	0.00	0.00	0.00	0.00	0.00	0.00
	Longma Country of Qujing	0.29	31.90	0.00	0.00	0.00	0.00	0.00	14.27	1.38
	Xuanwei City of Qujing	0.90	37.84	0.00	0.00	0.00	0.00	0.00	0.00	0.00
	Xuanwei City of Qujing	0.74	41.01	0.88	0.00	0.00	0.00	0.00	0.00	0.00
	Xuanwei City of Qujing	1.01	44.64	1.58	0.00	0.00	0.00	0.00	0.00	0.80
	Xuanwei City of Qujing	0.78	41.42	0.60	0.00	16.60	0.00	0.00	0.00	0.00
	Xuanwei City of Qujing	0.88	33.59	0.62	0.00	0.00	0.00	0.44	13.58	0.89
	Xuanwei City of Qujing	0.44	34.94	0.73	0.00	0.00	0.00	0.00	0.00	1.00
Northeastern Yunnan	Xundian Country of Kunming	0.86	39.37	1.14	0.00	0.00	0.00	0.33	18.68	0.83
	Xundian Country of Kunming	1.01	38.36	0.00	0.00	21.01	0.00	0.00	12.54	0.00
	Xundian Country of Kunming	0.86	33.85	0.00	0.34	17.46	0.00	1.02	11.19	0.00
	Xundian Country of Kunming	0.62	27.09	0.00	0.00	0.00	0.00	1.06	25.75	0.90

Xundian Country of Kunming	0.83	38.06	0.80	0.00	16.06	0.00	0.56	10.59	0.00
Xundian Country of Kunming	0.94	45.75	1.54	0.00	0.00	0.00	0.00	13.10	0.00
Xundian Country of Kunming	0.47	39.54	0.70	0.00	0.00	0.00	0.00	0.00	0.80
Xundian Country of Kunming	0.98	36.20	0.66	0.00	18.48	0.00	0.89	27.39	0.97
Xundian Country of Kunming	0.66	38.08	0.76	0.00	18.22	0.60	0.42	34.36	1.29
Xundian Country of Kunming	1.38	39.76	0.72	0.00	11.02	0.00	0.59	20.84	1.05
Luquan Country of Kunming	1.02	42.26	7.99	0.00	17.96	1.38	0.97	27.66	20.80
Luquan Country of Kunming	1.03	44.28	1.45	0.00	12.14	0.00	1.71	0.00	11.65
Luquan Country of Kunming	0.85	43.83	1.37	0.00	13.55	0.82	0.99	25.11	3.19
Luquan Country of Kunming	0.00	31.30	4.38	0.00	0.00	1.13	0.94	18.60	10.79
Luquan Country of Kunming	0.52	31.30	2.03	0.00	11.59	1.16	0.73	28.81	10.50
Luquan Country of Kunming	0.57	32.71	0.88	0.00	0.00	0.00	0.51	17.70	6.08
Luquan Country of Kunming	0.32	16.63	0.75	0.00	0.00	0.79	0.90	0.00	7.75
Luquan Country of Kunming	0.42	28.82	0.91	0.00	13.95	1.54	0.46	0.00	3.58
Luquan Country of Kunming	0.53	25.33	3.84	0.00	0.00	1.34	0.78	12.11	10.80
Luquan Country of Kunming	1.00	39.82	6.47	0.00	0.00	1.42	0.77	0.00	13.69
Luquan Country of Kunming	0.80	34.43	5.75	0.00	0.00	1.47	0.79	0.00	14.09
Dongchuan District of Kunming	0.96	26.56	1.07	0.37	0.00	0.00	1.09	13.24	6.50
Dongchuan District of Kunming	0.41	21.64	0.00	0.65	24.21	0.82	3.04	15.83	3.63
Dongchuan District of Kunming	1.25	38.84	1.21	0.30	15.87	0.00	2.72	27.88	1.81
Dongchuan District of Kunming	0.78	31.50	1.01	0.00	18.00	0.00	2.17	24.15	3.10
Dongchuan District of Kunming	1.53	36.38	1.20	0.42	12.79	0.00	3.02	27.42	2.66
Dongchuan District of Kunming	0.64	24.61	1.06	0.00	0.00	0.00	2.66	28.32	3.80
Dongchuan District of Kunming	1.26	35.36	1.77	0.38	12.92	0.90	2.62	30.91	17.68
Dongchuan District of Kunming	0.95	37.54	1.73	0.37	11.37	0.93	2.03	28.59	16.76
Dongchuan District of Kunming	0.87	28.36	0.00	0.00	0.00	0.00	0.73	12.33	3.23
Dongchuan District of Kunming	0.96	27.46	1.53	0.51	16.84	0.82	4.00	23.64	13.90

	Dongchuan District of Kunming	0.94	36.47	1.81	0.38	11.10	0.81	1.96	28.21	16.74
	Wuding Country of Kunming	0.62	32.35	3.07	0.00	0.00	0.00	0.53	10.67	2.57
	Wuding Country of Kunming	0.85	37.08	2.31	0.00	18.29	0.72	0.41	11.13	1.33
	Wuding Country of Kunming	0.92	42.51	2.45	0.00	11.72	0.00	0.00	12.77	0.60
	Wuding Country of Kunming	0.85	39.65	2.07	0.00	0.00	0.00	3.07	21.93	3.56
	Wuding Country of Kunming	1.19	43.42	2.05	0.00	0.00	0.00	0.44	12.35	1.91
	Wuding Country of Kunming	0.36	27.11	3.21	0.00	0.00	0.74	0.00	0.00	0.00
	Wuding Country of Kunming	1.16	38.06	3.00	0.00	0.00	0.00	0.00	0.00	0.78
	Wuding Country of Kunming	1.02	37.50	2.63	0.00	0.00	0.63	0.43	0.00	0.00
	Wuding Country of Kunming	2.08	54.92	4.36	0.47	15.42	0.00	1.35	13.41	1.26
	Qiaojia Country of Zhaotong	0.48	23.32	0.00	0.00	17.40	0.00	1.59	24.61	12.16
	Qiaojia Country of Zhaotong	1.33	60.48	1.13	0.62	24.43	0.60	4.46	22.46	4.72
	Qiaojia Country of Zhaotong	1.02	51.64	0.90	0.00	17.53	0.00	2.53	0.00	0.93
	Qiaojia Country of Zhaotong	0.94	38.01	0.71	0.42	21.77	0.00	4.19	22.39	0.69
	Qiaojia Country of Zhaotong	0.75	32.38	0.62	0.36	22.27	0.00	3.20	24.85	1.84
	Qiaojia Country of Zhaotong	1.75	56.60	0.89	0.38	18.20	0.00	2.69	28.04	1.87
	Qiaojia Country of Zhaotong	0.83	33.81	0.71	0.00	17.88	0.00	1.21	15.21	7.72
	Qiaojia Country of Zhaotong	1.20	63.55	1.09	0.47	32.76	0.00	2.79	24.96	1.66
	Qiaojia Country of Zhaotong	1.60	55.70	3.35	0.78	30.36	1.36	9.37	45.53	2.75
Northwestern Yunnan	Weixi Country of Diqing	0.68	45.52	1.19	0.00	11.48	0.00	0.70	49.93	6.45
	Weixi Country of Diqing	1.18	62.07	1.67	0.00	0.00	0.00	0.49	55.76	2.49
	Weixi Country of Diqing	1.31	67.90	1.82	0.00	16.37	0.69	0.48	56.07	2.26
	Weixi Country of Diqing	1.73	69.94	2.86	0.30	13.58	0.00	0.43	56.43	2.72
	Weixi Country of Diqing	1.10	56.08	1.38	0.00	0.00	0.00	0.30	48.74	1.69
	Weixi Country of Diqing	1.33	60.74	1.50	0.00	14.38	0.00	0.00	62.21	2.18
	Weixi Country of Diqing	0.00	34.00	0.66	0.00	20.47	0.66	0.39	51.27	1.67

Weixi Country of Diqing	0.65	53.14	1.53	0.00	16.61	0.00	0.36	60.80	4.10
Weixi Country of Diqing	0.51	37.03	1.04	0.00	0.00	0.00	0.00	22.57	0.82
Weixi Country of Diqing	0.00	53.18	1.47	0.00	18.59	0.74	0.37	45.18	5.20
Weixi Country of Diqing	1.31	62.98	1.10	0.00	12.92	0.62	0.67	25.93	1.51
Weixi Country of Diqing	0.70	45.48	1.25	0.00	0.00	0.00	0.69	57.16	2.51
Weixi Country of Diqing	0.64	42.87	1.68	0.00	0.00	0.61	0.60	54.87	4.09
Weixi Country of Diqing	0.97	50.82	1.62	0.29	19.19	0.68	0.35	41.52	1.50
Weixi Country of Diqing	1.49	75.93	1.85	0.00	11.26	0.74	0.61	64.32	3.96
Weixi Country of Diqing	0.64	57.59	1.35	0.00	15.61	0.68	0.44	47.44	1.92
Weixi Country of Diqing	0.00	0.00	0.00	0.83	63.73	1.58	0.74	59.54	4.11
Weixi Country of Diqing	0.65	57.23	1.08	0.00	0.00	0.72	0.73	56.46	3.38
Weixi Country of Diqing	1.17	57.86	1.46	0.00	11.79	0.72	0.96	45.95	3.19
Weixi Country of Diqing	0.98	45.19	1.87	0.00	10.95	0.62	0.50	51.66	2.60
Lanping Country of Nujiang	0.89	51.84	1.07	0.47	15.76	0.96	1.12	41.13	2.61
Lanping Country of Nujiang	0.67	55.31	0.00	0.00	16.32	0.00	0.46	21.15	0.00
Lanping Country of Nujiang	0.75	36.26	0.71	0.00	0.00	0.00	0.83	37.63	1.08
Lanping Country of Nujiang	1.60	66.40	1.10	0.00	0.00	0.00	0.89	40.99	0.67
Lanping Country of Nujiang	1.20	44.90	1.03	0.00	0.00	0.00	0.55	25.67	1.89
Lanping Country of Nujiang	0.56	48.30	0.00	0.00	0.00	0.00	0.00	18.58	0.61
Lanping Country of Nujiang	1.30	56.32	1.38	0.00	0.00	0.00	1.10	28.10	1.42
Lanping Country of Nujiang	1.70	66.08	0.92	0.00	0.00	0.00	0.61	24.70	0.97
Lanping Country of Nujiang	1.00	43.91	0.76	0.00	0.00	0.00	0.61	25.26	0.78
Lanping Country of Nujiang	0.48	14.64	0.00	0.00	0.00	0.00	0.55	13.28	0.93
Lanping Country of Nujiang	0.65	28.50	1.88	0.00	0.00	0.00	0.70	12.65	1.24
Lanping Country of Nujiang	0.90	43.82	1.27	0.00	0.00	0.00	0.35	17.51	1.13
Lanping Country of Nujiang	1.55	52.81	1.00	0.00	0.00	0.00	1.17	35.87	1.11
Lanping Country of Nujiang	0.66	27.89	0.76	0.00	0.00	0.00	0.71	19.64	1.27

Lanping Country of Nujiang	1.02	40.93	0.79	1.18	41.49	1.09	0.00	0.00	0.00
Lanping Country of Nujiang	0.69	39.79	0.00	0.00	0.00	0.00	0.66	21.55	1.02
Lushui Country of Nujiang	0.36	35.53	0.00	0.00	15.20	0.00	0.42	14.72	0.77
Lushui Country of Nujiang	0.61	37.69	0.00	0.00	0.00	0.00	0.42	0.00	0.00
Lushui Country of Nujiang	0.64	34.07	0.00	0.00	12.34	0.00	0.00	0.00	0.00
Lushui Country of Nujiang	0.39	30.41	0.00	0.00	0.00	0.00	0.32	0.00	0.00
Lushui Country of Nujiang	0.38	31.97	0.00	0.00	0.00	0.00	0.00	0.00	0.00
Lushui Country of Nujiang	0.50	40.58	0.00	0.00	0.00	0.61	0.00	0.00	0.00
Lushui Country of Nujiang	0.60	43.06	0.00	0.00	12.31	0.00	0.00	0.00	1.01
Lushui Country of Nujiang	0.00	27.70	0.00	0.00	12.25	0.00	0.00	0.00	0.00
Lushui Country of Nujiang	0.63	45.29	0.66	0.63	45.29	0.66	0.56	0.00	0.00
Lushui Country of Nujiang	0.55	35.50	0.00	0.00	0.00	0.00	0.60	39.34	1.07
Yulong Country of Lijiang	1.50	68.50	0.86	0.00	23.17	0.00	0.51	20.41	0.83
Yulong Country of Lijiang	1.28	59.41	0.78	0.00	0.00	0.00	0.00	0.00	0.00
Yulong Country of Lijiang	0.55	28.12	0.00	0.00	14.72	0.00	0.40	0.00	0.74
Yulong Country of Lijiang	1.48	58.68	1.26	0.00	16.16	0.00	0.53	20.25	0.98
Yulong Country of Lijiang	1.58	69.99	0.87	0.00	23.84	0.00	0.31	15.61	1.55
Yulong Country of Lijiang	1.56	67.41	0.94	0.00	32.06	0.00	0.66	26.88	0.86
Yulong Country of Lijiang	1.46	66.13	0.99	0.00	31.15	0.00	0.68	28.61	0.86
Yulong Country of Lijiang	1.77	60.00	0.00	0.00	22.21	0.00	0.44	0.00	1.09
Yulong Country of Lijiang	1.60	72.08	0.00	0.00	26.37	0.00	0.41	0.00	0.00
Yulong Country of Lijiang	0.65	49.52	0.00	0.00	28.21	0.60	0.31	15.71	0.79
Yulong Country of Lijiang	0.88	48.16	0.76	0.00	16.94	0.00	1.19	80.87	0.79
Yulong Country of Lijiang	0.42	16.02	1.21	0.00	28.97	0.00	0.00	13.53	0.00
Ninglang Country of Lijiang	1.74	76.84	10.09	0.47	55.50	2.99	2.29	60.10	1.98
Ninglang Country of Lijiang	0.67	44.46	0.63	0.36	23.72	0.00	0.96	26.92	0.93
Ninglang Country of Lijiang	0.81	57.81	0.94	0.00	16.44	0.00	2.08	40.56	1.63

	Ninglang Country of Lijiang	0.94	47.89	1.21	0.40	21.32	0.00	1.29	32.46	0.72
	Ninglang Country of Lijiang	0.48	42.47	0.74	0.31	16.67	0.00	1.21	43.30	0.70
	Ninglang Country of Lijiang	0.72	52.10	1.37	0.00	26.62	1.33	0.89	25.36	1.09
	Ninglang Country of Lijiang	0.00	18.47	0.00	1.05	39.06	1.17	1.43	26.22	0.76
	Ninglang Country of Lijiang	0.00	28.23	0.76	0.00	14.07	0.00	1.10	25.49	0.74
	Ninglang Country of Lijiang	0.67	42.38	0.93	0.54	20.56	0.00	0.88	30.60	0.82
	Ninglang Country of Lijiang	0.50	35.68	1.07	0.48	20.89	0.67	1.19	31.89	1.72
	Gucheng District of Lijiang	1.37	37.64	0.00	0.00	21.31	0.00	0.74	0.00	1.21
	Gucheng District of Lijiang	1.63	55.70	0.00	0.00	16.31	0.00	0.00	0.00	0.95
	Gucheng District of Lijiang	1.17	63.43	1.01	0.00	20.91	0.00	0.00	0.00	1.23
	Gucheng District of Lijiang	0.63	31.12	0.00	0.00	0.00	0.00	0.92	37.41	2.34
	Gucheng District of Lijiang	0.66	31.47	0.00	1.99	51.05	0.00	0.60	22.34	0.00
	Gucheng District of Lijiang	1.90	49.96	0.70	0.49	23.61	0.00	1.02	19.47	2.14
Western Yunnan	Tengchong Country of Baoshan	1.33	52.50	1.28	0.31	15.56	0.00	1.13	0.00	0.00
	Tengchong Country of Baoshan	1.12	56.52	2.61	0.00	0.00	0.00	0.46	21.56	0.00
	Tengchong Country of Baoshan	1.45	56.92	2.21	0.35	0.00	0.00	0.70	0.00	0.00
	Tengchong Country of Baoshan	1.61	53.96	1.64	0.44	0.00	0.00	1.30	18.94	1.16
	Tengchong Country of Baoshan	1.97	66.04	0.82	0.34	0.00	0.00	0.89	0.00	0.00
	Tengchong Country of Baoshan	1.31	42.39	1.23	0.00	0.00	0.00	0.68	0.00	0.00
	Tengchong Country of Baoshan	1.88	66.02	2.01	0.00	0.00	0.00	1.43	40.87	0.73
	Tengchong Country of Baoshan	1.72	66.83	1.21	0.43	16.74	0.00	1.28	32.52	0.00
	Tengchong Country of Baoshan	1.81	73.40	1.40	0.46	15.03	0.00	0.83	10.52	0.00
	Tengchong Country of Baoshan	1.74	60.82	1.49	0.55	13.42	0.60	1.43	35.23	0.62
	Longling Country of Baoshan	0.88	38.92	0.00	0.90	22.99	0.00	2.17	30.27	0.00
	Longling Country of Baoshan	1.07	43.39	0.00	0.44	13.58	0.00	0.61	12.31	0.83
	Longling Country of Baoshan	1.34	39.99	0.65	0.00	0.00	0.00	1.28	28.06	0.00
	Longling Country of Baoshan	1.31	55.91	0.98	0.33	11.59	0.00	0.69	19.73	0.60

Longling Country of Baoshan	1.16	44.91	0.00	0.70	0.00	0.00	1.09	13.93	0.00
Longling Country of Baoshan	0.76	38.96	0.00	0.57	0.00	0.00	1.13	10.74	0.00
Longling Country of Baoshan	1.46	45.95	0.94	0.34	0.00	0.00	1.26	0.00	0.00
Longling Country of Baoshan	0.77	36.20	0.00	0.60	12.59	0.00	1.59	25.82	0.00
Longling Country of Baoshan	1.23	48.05	1.10	0.00	10.58	0.00	0.85	17.69	0.61
Longling Country of Baoshan	0.85	39.09	0.00	0.37	0.00	0.00	0.91	0.00	0.00
Changning Country of Baoshan	1.42	48.52	1.89	0.62	11.05	0.00	1.29	0.00	0.00
Changning Country of Baoshan	1.36	52.47	0.92	0.78	22.69	0.00	1.90	0.00	0.00
Changning Country of Baoshan	1.19	41.44	1.06	0.00	0.00	0.00	1.12	0.00	0.00
Changning Country of Baoshan	1.10	44.08	0.00	0.56	15.21	0.00	0.00	0.00	0.00
Changning Country of Baoshan	0.73	34.57	0.00	0.67	14.27	0.00	1.48	0.00	0.00
Changning Country of Baoshan	1.47	45.07	1.36	0.46	0.00	0.00	1.49	0.00	0.00
Changning Country of Baoshan	1.18	49.43	0.97	1.37	0.00	0.00	0.60	15.44	0.00
Changning Country of Baoshan	0.51	0.00	0.00	0.90	35.81	0.00	1.05	0.00	0.00
Changning Country of Baoshan	1.36	48.07	0.00	0.35	12.37	0.00	1.56	0.00	0.00
Changning Country of Baoshan	1.48	53.97	1.06	0.97	19.21	0.00	2.34	19.71	0.00
Binchuan Country of Dali	0.72	35.37	1.44	0.69	23.65	0.65	1.69	32.91	1.68
Binchuan Country of Dali	0.00	19.14	0.61	0.40	16.21	0.00	2.09	44.08	1.52
Binchuan Country of Dali	0.00	36.15	1.14	0.40	20.22	0.64	1.49	28.78	1.30
Binchuan Country of Dali	0.00	19.81	0.00	0.54	14.97	0.00	1.95	30.69	3.17
Binchuan Country of Dali	0.00	19.43	2.35	0.31	17.65	0.92	2.29	47.60	2.10
Binchuan Country of Dali	0.00	21.20	0.00	0.40	0.00	0.00	1.66	27.22	3.68
Binchuan Country of Dali	0.45	29.13	1.17	0.45	21.37	0.96	2.03	48.77	1.88
Binchuan Country of Dali	0.00	18.03	1.27	0.41	25.86	2.20	2.10	44.00	2.41
Binchuan Country of Dali	0.75	36.72	1.41	0.71	38.79	1.47	1.90	27.35	3.43
Binchuan Country of Dali	0.34	26.88	2.54	0.00	12.87	0.72	1.96	40.34	1.77
Binchuan Country of Dali	0.00	16.93	2.05	0.00	12.58	0.65	2.31	48.70	2.06

Dali Cangshan Global Geopark	1.14	51.67	0.00	0.66	28.85	0.00	2.03	23.22	0.00
Dali Cangshan Global Geopark	0.83	41.74	0.00	1.03	45.97	0.70	1.20	34.18	1.11
Dali Cangshan Global Geopark	0.85	48.18	0.00	0.41	22.53	0.00	1.74	19.49	0.00
Dali Cangshan Global Geopark	1.17	57.18	0.76	0.53	39.41	0.00	2.11	28.40	0.65
Dali Cangshan Global Geopark	1.21	49.63	0.00	0.53	22.41	0.00	1.86	19.13	0.00
Dali Cangshan Global Geopark	0.76	41.57	0.00	0.41	19.98	0.00	2.13	31.09	0.71
Dali Cangshan Global Geopark	1.34	60.58	1.03	0.65	20.17	0.00	1.99	14.60	0.00
Dali Cangshan Global Geopark	1.14	54.87	0.00	0.73	26.33	0.00	2.96	33.19	0.83
Dali Cangshan Global Geopark	1.09	58.03	1.80	0.75	50.18	0.87	2.06	43.74	1.19
Dali Cangshan Global Geopark	1.31	59.43	1.34	0.73	35.59	0.68	1.52	18.93	0.64
Er'hai Country of Dali	1.04	61.09	0.87	1.14	39.87	0.00	2.65	11.71	0.73
Er'hai Country of Dali	1.31	52.82	1.18	0.71	28.17	0.67	2.47	39.29	1.56
Er'hai Country of Dali	1.73	67.88	1.37	0.42	32.14	0.00	2.50	45.22	1.74
Er'hai Country of Dali	1.11	51.37	0.00	0.80	32.26	0.00	2.73	47.43	1.24
Er'hai Country of Dali	1.09	39.59	3.36	0.99	44.57	1.26	2.52	60.56	3.36
Er'hai Country of Dali	1.19	50.19	1.81	0.93	31.18	0.70	2.91	46.35	0.74
Er'hai Country of Dali	1.47	56.19	1.57	0.90	48.78	1.06	2.88	65.96	2.86
Er'hai Country of Dali	1.72	60.23	1.20	0.91	32.24	0.63	2.90	49.68	1.37
Er'hai Country of Dali	1.16	58.09	1.45	0.66	28.10	0.00	1.94	40.21	0.00
Er'hai Country of Dali	1.33	61.01	0.72	0.79	37.69	0.00	2.85	47.65	1.28
Heqing Country of Dali	1.13	48.50	1.75	0.00	18.31	0.00	0.59	27.04	2.04
Heqing Country of Dali	1.35	48.48	0.89	0.00	15.62	0.00	0.38	20.51	1.61
Heqing Country of Dali	0.92	45.53	1.49	0.00	17.96	0.00	0.00	24.18	1.61
Heqing Country of Dali	1.05	47.55	1.40	0.00	31.77	0.75	0.78	27.68	1.44
Heqing Country of Dali	0.94	55.86	1.03	0.00	16.30	0.00	0.31	20.36	1.36
Heqing Country of Dali	0.96	53.70	0.86	0.00	15.00	0.00	0.51	24.54	1.40
Heqing Country of Dali	0.77	50.12	0.71	0.00	15.78	0.00	0.00	28.51	1.05

	Heqing Country of Dali	1.21	59.50	0.00	0.00	25.02	0.00	0.00	16.02	1.16
	Heqing Country of Dali	0.51	38.58	0.00	0.00	24.31	0.00	0.00	35.96	2.00
	Heqing Country of Dali	0.84	41.64	0.00	0.00	12.07	0.00	0.64	26.56	1.14
	Yongping Country of Dali	0.96	37.71	0.89	0.00	0.00	0.00	0.62	20.03	1.37
	Yongping Country of Dali	1.51	55.29	1.01	0.00	0.00	0.00	0.29	0.00	1.37
	Yongping Country of Dali	0.65	43.05	0.89	0.00	15.34	0.64	0.31	0.00	1.20
	Yongping Country of Dali	0.52	45.88	1.89	0.00	0.00	0.00	0.46	26.07	1.48
	Yongping Country of Dali	0.82	48.94	0.84	0.00	0.00	0.00	0.41	0.00	0.93
	Yongping Country of Dali	0.64	34.06	0.63	0.00	18.24	0.00	0.53	26.42	2.68
	Yongping Country of Dali	0.87	41.93	1.40	0.00	12.47	0.00	0.52	14.35	1.76
	Yongping Country of Dali	0.44	33.06	0.98	0.00	0.00	0.00	0.00	0.00	1.14
	Yongping Country of Dali	0.70	42.31	1.44	0.00	0.00	0.00	0.58	0.00	1.36
	Yongping Country of Dali	0.40	31.73	0.75	0.00	15.44	0.00	0.00	0.00	0.71
	Nanjian Country of Dali	1.27	57.70	4.53	0.53	36.13	0.87	1.36	42.89	0.77
	Nanjian Country of Dali	2.18	80.39	5.31	0.00	27.88	0.00	1.56	55.87	0.94
	Nanjian Country of Dali	1.44	57.26	6.94	0.60	32.05	2.97	1.93	61.67	6.51
	Nanjian Country of Dali	1.62	61.80	6.01	0.94	37.04	0.87	1.40	45.30	1.63
	Nanjian Country of Dali	1.26	48.81	3.23	0.69	36.26	1.13	1.77	35.93	1.52
	Nanjian Country of Dali	1.45	50.74	5.21	0.00	18.81	0.00	0.86	37.08	0.00
	Nanjian Country of Dali	1.76	55.20	1.85	0.71	0.00	0.63	1.32	15.82	1.09
	Nanjian Country of Dali	1.65	44.02	2.16	0.66	0.00	0.00	2.15	11.26	1.96
	Nanjian Country of Dali	1.41	48.36	3.72	1.03	0.00	0.00	1.88	12.82	0.00
	Nanjian Country of Dali	1.30	46.66	2.68	0.34	0.00	0.72	1.43	19.42	1.98
Southeastern Yunnan	Luxi Country of Honghe	0.32	30.48	0.00	0.00	0.00	0.00	0.00	0.00	0.00
	Luxi Country of Honghe	0.64	34.44	0.00	0.00	0.00	0.00	0.00	16.26	0.00
	Luxi Country of Honghe	0.70	35.76	1.08	0.00	0.00	0.00	0.00	0.00	0.00

Luxi Country of Honghe	0.76	36.68	0.00	0.00	12.37	0.00	1.20	20.87	1.09
Luxi Country of Honghe	0.31	28.75	0.97	0.00	0.00	0.00	0.00	0.00	0.00
Luxi Country of Honghe	0.34	27.90	0.00	0.00	14.42	0.00	0.00	0.00	0.00
Luxi Country of Honghe	0.00	27.08	0.00	0.00	0.00	0.00	0.00	0.00	0.00
Luxi Country of Honghe	0.40	26.88	0.00	0.00	0.00	0.00	0.00	0.00	0.00
Luxi Country of Honghe	0.56	40.65	0.68	0.00	12.64	0.00	0.00	0.00	0.00
Luxi Country of Honghe	0.39	39.27	0.74	0.00	0.00	0.00	0.00	0.00	0.00
Mile Country of Honghe	1.28	45.62	0.99	0.00	24.72	0.00	0.68	41.12	5.26
Mile Country of Honghe	1.17	43.77	0.63	0.00	0.00	0.00	0.41	0.00	0.00
Mile Country of Honghe	1.02	37.35	0.00	0.00	0.00	0.00	0.32	0.00	0.00
Mile Country of Honghe	1.51	44.81	0.96	0.00	0.00	0.00	0.44	0.00	0.00
Mile Country of Honghe	0.80	35.55	0.00	0.00	0.00	0.00	0.33	0.00	0.00
Mile Country of Honghe	0.86	30.96	0.00	0.00	0.00	0.73	0.72	13.73	0.70
Mile Country of Honghe	0.89	37.55	0.68	0.00	14.53	0.00	1.20	27.26	0.95
Mile Country of Honghe	1.16	37.87	0.81	0.00	0.00	0.00	0.63	0.00	0.00
Mile Country of Honghe	1.80	47.67	1.29	0.54	0.00	0.00	1.82	0.00	0.77
Mile Country of Honghe	0.67	34.69	0.00	0.00	0.00	0.00	0.00	0.00	0.00
Mengzi Country of Honghe	0.37	22.99	0.00	0.00	11.97	0.00	0.54	19.47	0.00
Mengzi Country of Honghe	0.78	39.99	3.73	0.00	0.00	0.00	0.00	0.00	0.62
Mengzi Country of Honghe	0.48	29.20	0.00	0.00	0.00	0.00	0.35	0.00	0.00
Mengzi Country of Honghe	0.38	27.13	0.00	0.00	0.00	0.00	0.92	0.00	0.00
Mengzi Country of Honghe	0.68	37.79	0.00	0.00	0.00	0.00	0.88	12.34	0.69
Mengzi Country of Honghe	0.00	21.35	0.00	0.00	0.00	0.00	0.53	0.00	1.32
Mengzi Country of Honghe	0.51	34.87	0.00	0.00	0.00	0.00	1.13	16.07	1.76
Mengzi Country of Honghe	0.00	18.03	0.00	0.00	14.68	0.00	0.79	18.42	2.08
Mengzi Country of Honghe	0.64	41.40	0.00	0.00	11.44	0.00	0.85	0.00	0.00
Mengzi Country of Honghe	0.32	27.37	0.00	0.00	0.00	0.00	0.88	0.00	0.00

Pingbian Country of Honghe	1.18	38.73	1.11	1.00	14.52	0.72	1.32	12.02	1.46
Pingbian Country of Honghe	0.98	32.06	1.09	0.00	0.00	0.00	0.42	30.71	0.78
Pingbian Country of Honghe	0.96	35.58	0.00	0.36	0.00	0.00	0.79	0.00	0.00
Pingbian Country of Honghe	1.01	26.50	0.83	0.00	0.00	0.00	0.83	13.73	1.74
Pingbian Country of Honghe	0.63	20.64	0.00	0.00	0.00	0.00	0.00	0.00	0.00
Pingbian Country of Honghe	0.86	31.35	0.00	0.00	0.00	0.00	0.00	0.00	0.00
Pingbian Country of Honghe	0.60	15.51	0.71	0.00	0.00	0.00	0.86	13.71	2.01
Pingbian Country of Honghe	0.93	25.90	0.61	0.00	0.00	0.00	0.00	0.00	0.00
Pingbian Country of Honghe	1.33	36.10	0.80	0.00	0.00	0.00	0.31	19.85	0.74
Pingbian Country of Honghe	1.10	33.26	0.00	0.00	0.00	0.00	0.38	0.00	0.00
Gejiu City of Honghe	0.70	39.88	0.00	0.00	0.00	0.00	0.00	11.12	0.00
Gejiu City of Honghe	0.55	34.40	0.00	0.00	0.00	0.00	0.00	0.00	0.00
Gejiu City of Honghe	1.03	43.85	0.00	0.00	0.00	0.00	0.43	0.00	0.00
Gejiu City of Honghe	0.71	32.12	0.00	0.00	0.00	0.00	0.57	0.00	0.00
Gejiu City of Honghe	0.62	30.66	0.00	0.00	0.00	0.00	0.00	0.00	0.00
Gejiu City of Honghe	0.62	31.67	0.00	0.00	11.69	0.00	0.78	27.27	0.00
Gejiu City of Honghe	0.64	31.08	0.00	0.35	0.00	0.00	0.63	0.00	0.00
Gejiu City of Honghe	0.75	35.08	0.00	0.00	0.00	0.00	0.29	0.00	0.00
Gejiu City of Honghe	1.32	55.72	0.00	0.00	0.00	0.00	0.90	11.23	0.00
Honghe Country of Honghe	0.85	44.98	0.00	0.00	15.53	0.00	0.30	30.22	1.05
Honghe Country of Honghe	1.35	49.18	0.73	0.00	16.84	0.00	0.81	14.10	0.97
Honghe Country of Honghe	1.79	60.28	1.04	0.48	18.56	0.00	0.56	0.00	0.00
Honghe Country of Honghe	1.11	46.92	1.50	0.00	0.00	0.00	0.91	14.53	0.75
Honghe Country of Honghe	0.96	42.48	0.00	0.00	13.05	0.00	0.72	0.00	0.00
Honghe Country of Honghe	0.93	49.61	0.73	0.00	0.00	0.00	0.62	0.00	0.86
Honghe Country of Honghe	0.53	33.19	0.00	0.00	16.19	0.63	0.83	0.00	0.68
Honghe Country of Honghe	1.05	39.47	0.71	0.00	0.00	0.00	0.46	0.00	0.00

Honghe Country of Honghe	0.64	38.57	0.62	0.00	0.00	0.00	0.52	0.00	0.89
Qiubei Country of Wenshan	0.91	33.05	0.00	0.36	0.00	0.00	0.77	0.00	0.00
Qiubei Country of Wenshan	0.94	42.10	0.00	0.00	0.00	0.00	0.40	0.00	0.00
Qiubei Country of Wenshan	1.18	34.54	1.07	0.40	13.47	0.00	0.57	11.69	1.00
Qiubei Country of Wenshan	0.85	28.63	0.00	0.00	0.00	0.00	1.50	18.13	1.34
Qiubei Country of Wenshan	0.35	30.16	0.00	0.00	0.00	0.00	0.00	0.00	0.00
Qiubei Country of Wenshan	0.49	41.77	0.00	0.00	0.00	0.00	0.42	0.00	0.00
Qiubei Country of Wenshan	0.40	26.78	0.00	0.00	0.00	0.00	1.68	0.00	2.59
Qiubei Country of Wenshan	1.69	58.73	0.00	0.00	0.00	0.00	0.00	0.00	0.00
Qiubei Country of Wenshan	0.32	28.70	0.00	0.00	0.00	0.00	0.49	0.00	0.00
Qiubei Country of Wenshan	0.47	35.41	0.00	0.00	0.00	0.00	0.32	11.73	1.35
Qiubei Country of Wenshan	0.00	23.53	0.00	0.00	0.00	0.00	0.00	0.00	0.00
Qiubei Country of Wenshan	0.00	22.10	0.00	0.00	0.00	0.00	0.00	0.00	0.66
Qiubei Country of Wenshan	0.36	35.67	0.00	0.00	0.00	0.00	0.00	0.00	1.20
Qiubei Country of Wenshan	0.00	29.65	0.63	0.00	0.00	0.00	0.00	0.00	1.25
Qiubei Country of Wenshan	0.98	51.11	0.00	0.00	0.00	0.00	0.55	0.00	0.00
Qiubei Country of Wenshan	1.22	52.41	3.05	0.42	0.00	0.00	0.59	0.00	0.62
Qiubei Country of Wenshan	1.08	47.75	0.68	0.31	18.94	0.76	0.00	0.00	1.65
Qiubei Country of Wenshan	0.00	0.00	0.00	0.66	0.00	0.65	0.97	45.37	0.84
Qiubei Country of Wenshan	0.00	0.00	0.00	0.56	0.00	0.68	1.04	44.58	3.57
Yanshan Country of Wenshan	0.92	38.97	0.00	0.00	0.00	0.00	0.42	0.00	0.00
Yanshan Country of Wenshan	0.41	25.54	0.00	0.00	10.53	0.00	0.00	0.00	0.00
Yanshan Country of Wenshan	0.70	37.08	0.00	0.00	0.00	0.00	0.00	0.00	0.00
Yanshan Country of Wenshan	0.55	28.47	0.00	0.00	0.00	0.00	0.00	0.00	0.00
Yanshan Country of Wenshan	0.38	21.94	0.00	0.00	12.47	0.00	0.00	0.00	0.00
Yanshan Country of Wenshan	0.75	39.41	0.00	0.00	0.00	0.00	0.00	0.00	0.00
Yanshan Country of Wenshan	0.88	34.89	0.94	0.00	0.00	0.00	0.00	0.00	0.00

	Yanshan Country of Wenshan	0.89	27.54	0.00	0.00	11.48	0.00	0.71	10.59	0.72
	Yanshan Country of Wenshan	0.44	34.71	0.00	0.00	11.80	0.00	0.00	0.00	0.00
	Yanshan Country of Wenshan	0.59	27.96	0.00	0.00	13.22	0.00	0.00	10.81	0.68
	Wenshan City of Wenshan	0.76	34.13	0.00	0.00	0.00	0.00	0.94	11.09	0.00
	Wenshan City of Wenshan	0.67	40.42	0.00	0.00	0.00	0.00	0.52	0.00	0.00
	Wenshan City of Wenshan	0.67	34.80	0.62	0.00	0.00	0.00	0.00	0.00	0.00
	Wenshan City of Wenshan	0.89	30.71	0.00	0.00	0.00	0.00	0.49	0.00	0.00
	Wenshan City of Wenshan	0.34	18.67	0.00	0.00	0.00	0.00	0.31	0.00	0.00
	Wenshan City of Wenshan	0.55	35.93	0.00	0.00	0.00	0.00	0.44	0.00	1.07
	Wenshan City of Wenshan	0.74	29.25	0.00	0.00	0.00	0.00	0.49	0.00	0.00
	Wenshan City of Wenshan	1.19	39.29	1.52	0.00	0.00	0.00	0.30	0.00	0.00
	Wenshan City of Wenshan	0.62	27.43	0.00	0.45	13.81	0.00	1.47	13.87	1.28
	Wenshan City of Wenshan	1.00	37.69	0.61	0.00	0.00	0.00	2.00	53.91	1.99
Southwestern Yunnan	Jingdong Country of Pu'er	1.70	42.98	0.00	0.69	18.30	0.00	1.34	41.04	0.00
	Jingdong Country of Pu'er	1.05	43.13	0.00	0.50	18.34	0.00	1.34	13.46	0.00
	Jingdong Country of Pu'er	0.87	34.38	0.00	0.70	12.69	0.00	0.72	0.00	0.00
	Jingdong Country of Pu'er	0.88	43.38	0.00	0.76	13.53	0.00	0.85	19.54	0.00
	Jingdong Country of Pu'er	1.03	38.74	0.00	0.00	0.00	0.00	0.97	22.13	0.00
	Jingdong Country of Pu'er	1.06	49.63	0.00	0.40	0.00	0.00	0.65	0.00	0.00
	Jingdong Country of Pu'er	1.51	38.87	0.00	0.69	10.64	0.00	1.82	11.61	0.00
	Jingdong Country of Pu'er	1.46	46.70	0.00	0.42	0.00	0.00	0.34	0.00	0.00
	Jingdong Country of Pu'er	1.43	45.54	0.00	0.66	18.25	0.00	1.00	12.26	0.00
	Jingdong Country of Pu'er	0.89	40.47	0.00	0.48	15.40	0.00	0.60	0.00	0.00
	Zhenyuan Country of Pu'er	1.04	54.51	1.41	0.00	13.79	0.71	2.12	25.40	4.26
	Zhenyuan Country of Pu'er	0.34	20.07	0.00	0.00	15.90	0.00	1.01	21.87	0.00
	Zhenyuan Country of Pu'er	0.56	32.81	0.00	0.31	11.62	0.00	1.13	15.14	0.00

	Zhenyuan Country of Pu'er	0.46	37.36	0.00	0.00	0.00	0.00	2.26	0.00	0.00
	Zhenyuan Country of Pu'er	0.48	21.00	0.00	0.00	0.00	0.00	1.35	22.12	0.65
	Zhenyuan Country of Pu'er	0.56	31.83	0.70	0.00	12.23	0.00	1.29	22.24	1.18
	Zhenyuan Country of Pu'er	0.71	19.59	0.00	0.42	11.20	0.00	1.24	18.14	1.46
	Zhenyuan Country of Pu'er	0.85	36.20	0.91	0.00	10.59	0.00	0.93	0.00	0.00
	Zhenyuan Country of Pu'er	1.12	44.37	0.86	0.67	10.95	0.00	2.91	0.00	3.14
	Zhenyuan Country of Pu'er	0.69	36.00	1.19	0.00	12.53	0.00	1.43	19.93	3.39
	Mojiang Country of Pu'er	1.01	35.28	0.00	0.72	13.40	0.00	1.63	20.90	0.00
	Mojiang Country of Pu'er	1.67	53.99	0.66	0.36	15.65	0.00	1.81	10.57	0.00
	Mojiang Country of Pu'er	0.98	41.69	0.00	0.00	11.93	0.00	1.69	21.79	0.00
	Mojiang Country of Pu'er	1.36	38.04	0.61	0.52	13.25	0.00	1.58	19.98	0.00
	Mojiang Country of Pu'er	1.27	48.90	0.00	0.54	14.84	0.00	1.67	22.68	0.00
	Mojiang Country of Pu'er	0.95	39.55	0.00	0.55	0.00	0.00	1.25	0.00	0.97
	Mojiang Country of Pu'er	1.64	51.58	0.75	0.77	18.99	0.00	1.56	25.30	0.00
	Mojiang Country of Pu'er	1.15	42.13	0.61	0.63	13.64	0.00	0.65	0.00	0.00
	Mojiang Country of Pu'er	1.27	41.51	0.00	0.53	12.17	0.00	1.42	18.73	0.00
	Mojiang Country of Pu'er	1.14	39.73	0.00	0.43	14.42	0.00	1.94	23.45	0.87
Guizhou	Qishe Town of Xingyi	0.93	33.43	0.00	1.20	10.68	0.00	1.40	0.00	0.00
	Qishe Town of Xingyi	1.23	33.53	0.00	0.56	0.00	1.67	1.13	11.38	0.00
	Qishe Town of Xingyi	1.32	36.34	0.00	0.62	0.00	0.00	1.54	17.07	0.00
	Qishe Town of Xingyi	1.08	36.57	0.00	0.36	0.00	0.00	1.82	29.61	0.00
	Qishe Town of Xingyi	1.01	27.10	0.00	0.33	0.00	0.00	1.14	0.00	0.00
	Qishe Town of Xingyi	0.76	30.66	0.00	0.35	0.00	0.00	1.03	0.00	0.00
	Qishe Town of Xingyi	0.84	30.67	0.00	0.55	0.00	0.00	1.27	15.45	0.00
	Qishe Town of Xingyi	1.05	39.59	0.98	0.80	0.00	0.00	1.90	18.91	0.00
	Qishe Town of Xingyi	1.24	40.64	0.81	1.28	14.05	0.00	1.75	19.78	0.00
	Qishe Town of Xingyi	0.79	39.84	0.00	0.67	0.00	0.00	1.73	17.68	0.00

Baiwanyao Town of Xingyi	0.00	29.96	1.00	0.00	0.00	4.44	0.00	0.00	0.00
Baiwanyao Town of Xingyi	0.00	19.24	0.62	0.00	13.22	0.00	0.35	0.00	0.00
Baiwanyao Town of Xingyi	0.43	27.47	0.84	0.00	0.00	0.00	0.97	0.00	0.00
Baiwanyao Town of Xingyi	0.00	22.19	0.60	0.00	0.00	0.00	0.42	16.53	0.00
Baiwanyao Town of Xingyi	0.00	25.71	0.70	0.00	0.00	0.00	0.38	13.12	0.00
Baiwanyao Town of Xingyi	0.00	22.82	0.00	0.00	15.72	0.00	0.00	0.00	0.00
Baiwanyao Town of Xingyi	0.39	28.79	0.80	0.00	17.78	0.00	0.29	34.86	0.00
Baiwanyao Town of Xingyi	0.00	31.98	0.89	0.00	12.14	0.00	0.00	0.00	0.00
Baiwanyao Town of Xingyi	0.00	28.12	0.70	0.00	12.13	0.00	0.00	0.00	0.00
Baiwanyao Town of Xingyi	0.35	25.50	0.93	0.00	0.00	0.00	0.31	0.00	0.00
Baiwanyao Town of Xingyi	0.38	27.60	1.02	0.00	0.00	0.00	0.31	0.00	0.00
Sala Town of Bijie	0.79	34.06	1.03	0.46	11.22	0.00	1.61	12.21	0.00
Sala Town of Bijie	0.68	38.93	0.00	0.36	18.82	0.00	0.83	0.00	0.00
Sala Town of Bijie	1.12	33.92	1.30	0.33	0.00	0.00	1.24	11.53	0.83
Sala Town of Bijie	1.53	44.83	1.81	0.36	0.00	0.00	1.33	20.50	0.00
Sala Town of Bijie	1.17	34.23	0.95	0.45	0.00	0.00	1.76	19.36	0.00
Sala Town of Bijie	1.65	49.59	0.97	0.85	13.89	0.00	1.68	10.76	0.00
Sala Town of Bijie	0.92	42.94	0.00	0.73	20.35	0.00	0.93	0.00	0.00
Sala Town of Bijie	0.58	27.48	0.69	0.59	15.88	0.00	1.01	0.00	0.00
Sala Town of Bijie	0.83	43.20	1.09	0.59	0.00	0.00	0.62	0.00	0.00
Sala Town of Bijie	0.98	42.02	0.00	0.73	21.02	0.00	1.61	0.00	0.00
Dafang Country of Bijie	1.16	44.74	2.14	0.44	15.44	0.90	0.33	0.00	0.66
Dafang Country of Bijie	1.67	55.22	0.00	0.48	14.25	0.00	0.52	0.00	0.00
Dafang Country of Bijie	1.02	49.83	0.00	0.71	13.23	0.00	0.74	10.70	1.63
Dafang Country of Bijie	1.11	37.47	1.03	0.59	17.45	0.66	0.45	0.00	0.00
Dafang Country of Bijie	0.89	39.97	0.00	0.38	0.00	0.00	0.34	0.00	0.00
Dafang Country of Bijie	1.01	42.73	0.00	0.00	0.00	0.00	0.95	0.00	1.10

Dafang Country of Bijie	0.99	31.96	0.88	0.31	0.00	0.00	0.46	0.00	0.00
Dafang Country of Bijie	1.02	35.58	0.67	0.67	14.98	0.00	0.70	0.00	0.76
Dafang Country of Bijie	0.90	36.05	0.00	0.55	0.00	0.00	0.38	0.00	0.00
Qianxi Country of Bijie	0.94	42.94	0.00	0.59	0.00	0.00	1.11	0.00	0.79
Qianxi Country of Bijie	0.72	36.18	0.00	0.33	0.00	0.00	0.37	0.00	0.00
Qianxi Country of Bijie	0.75	35.25	0.00	0.00	14.37	0.00	0.46	15.73	0.00
Qianxi Country of Bijie	0.86	33.67	0.00	0.55	14.20	0.00	0.93	0.00	0.00
Qianxi Country of Bijie	0.88	38.80	0.00	0.31	0.00	0.00	0.87	0.00	0.73
Qianxi Country of Bijie	1.18	41.99	0.00	0.38	0.00	0.00	0.56	0.00	0.00
Qianxi Country of Bijie	0.86	40.17	0.00	0.00	0.00	0.00	0.98	0.00	0.98
Qianxi Country of Bijie	0.89	41.17	0.00	0.00	0.00	0.00	0.48	0.00	0.00
Qianxi Country of Bijie	1.00	43.27	0.00	0.36	20.01	0.00	1.02	12.21	0.00
Zhongshan District of Anshun	0.75	32.73	0.99	0.39	14.05	0.00	1.98	16.93	0.73
Zhongshan District of Anshun	1.04	48.93	0.94	0.50	16.02	0.87	3.48	20.65	2.48
Zhongshan District of Anshun	1.40	51.85	1.33	0.75	21.90	0.81	2.55	17.34	1.27
Zhongshan District of Anshun	1.68	48.56	0.88	0.51	19.24	0.73	2.01	21.77	3.49
Zhongshan District of Anshun	1.75	46.44	4.08	0.81	19.71	0.90	5.17	23.16	2.58
Zhongshan District of Anshun	1.42	52.23	1.60	0.45	20.77	0.00	2.25	19.33	3.95
Zhongshan District of Anshun	1.23	49.74	1.50	0.42	14.12	0.70	2.15	15.41	2.57
Zhongshan District of Anshun	0.97	35.83	0.75	0.62	20.49	1.63	3.99	17.75	4.49
Zhongshan District of Anshun	0.67	31.68	0.87	0.48	16.75	1.52	4.13	31.12	5.15
Zhongshan District of Anshun	1.20	49.45	1.77	1.31	27.92	0.79	4.19	12.73	1.57
Guanling Country of Anshun	0.50	40.39	0.78	0.00	0.00	0.00	0.00	0.00	0.00
Guanling Country of Anshun	0.94	39.32	2.11	0.00	0.00	0.00	0.00	0.00	0.00
Guanling Country of Anshun	0.58	36.31	0.86	0.00	0.00	0.00	0.00	0.00	0.00
Guanling Country of Anshun	0.87	39.04	1.03	0.00	0.00	0.00	0.00	0.00	0.00
Guanling Country of Anshun	0.73	43.13	0.88	0.00	0.00	0.00	0.00	0.00	0.00

Guanling Country of Anshun	0.58	42.83	1.30	0.00	0.00	0.00	0.00	0.00	0.00
Guanling Country of Anshun	0.37	35.10	0.00	0.00	0.00	0.00	0.38	0.00	0.67
Guanling Country of Anshun	0.00	30.20	0.69	0.00	0.00	0.00	0.00	0.00	0.00
Guanling Country of Anshun	0.89	37.54	1.25	0.00	0.00	0.00	0.00	0.00	0.00
Guanling Country of Anshun	0.55	33.86	0.63	0.00	12.09	0.00	0.00	0.00	0.00
Kaiyang Country of Guiyang	1.72	46.61	0.82	0.34	9.24	0.00	2.24	25.34	1.50
Kaiyang Country of Guiyang	1.57	23.28	0.00	0.00	0.00	0.00	1.27	15.37	2.06
Kaiyang Country of Guiyang	1.07	41.49	0.00	0.29	0.00	0.00	0.91	0.00	0.00
Kaiyang Country of Guiyang	1.84	53.29	0.76	0.56	10.52	0.00	2.26	20.71	1.49
Kaiyang Country of Guiyang	1.43	43.23	0.00	0.00	4.35	0.00	1.03	0.00	0.00
Kaiyang Country of Guiyang	1.11	40.41	0.00	0.30	4.47	0.00	0.83	3.07	0.00
Kaiyang Country of Guiyang	1.45	35.08	0.71	0.00	10.36	0.00	0.78	0.00	0.00
Kaiyang Country of Guiyang	1.48	46.91	1.01	0.51	5.67	0.00	2.24	4.67	1.54
Kaiyang Country of Guiyang	1.62	55.56	1.09	0.45	1.31	0.00	0.95	2.98	0.00
Kaiyang Country of Guiyang	1.87	54.40	0.61	0.40	14.24	0.96	0.76	0.00	1.44
Zhanjie Town of Qingzhen	1.43	41.19	0.00	0.61	15.99	0.00	1.33	24.49	0.00
Zhanjie Town of Qingzhen	1.03	33.96	0.00	0.34	7.11	0.00	1.87	28.58	2.96
Zhanjie Town of Qingzhen	0.90	33.49	0.00	0.00	4.55	0.00	1.04	9.37	1.41
Zhanjie Town of Qingzhen	1.39	34.26	3.47	0.00	2.95	0.00	1.24	24.30	4.48
Zhanjie Town of Qingzhen	0.92	25.27	0.00	0.45	4.86	0.00	0.49	1.26	0.94
Zhanjie Town of Qingzhen	1.00	33.17	0.00	0.00	3.23	0.00	0.90	5.18	0.00
Zhanjie Town of Qingzhen	1.09	44.84	0.00	0.45	6.62	0.00	0.40	1.16	0.00
Zhanjie Town of Qingzhen	1.63	37.66	0.96	0.31	4.44	0.87	1.61	4.55	1.30
Zhanjie Town of Qingzhen	1.26	40.42	0.00	0.00	1.51	0.00	0.92	1.51	0.00
Zhanjie Town of Qingzhen	1.20	42.24	1.29	0.00	1.74	0.00	0.38	2.97	0.94
Longli Country of Douyun	1.23	45.42	0.93	1.22	31.73	0.00	1.35	24.89	0.00
Longli Country of Douyun	1.45	47.50	0.00	0.37	21.55	0.00	1.39	44.36	0.00

Longli Country of Douyun	1.27	52.23	0.63	0.33	30.62	0.00	0.77	15.33	0.00
Longli Country of Douyun	0.94	42.13	0.00	0.48	13.92	0.00	0.82	15.23	0.65
Longli Country of Douyun	0.96	39.30	0.00	0.60	20.95	0.00	1.28	20.12	0.98
Longli Country of Douyun	1.10	39.86	1.78	0.68	26.55	0.75	1.65	33.67	2.35
Longli Country of Douyun	1.13	39.52	0.00	0.81	13.43	0.00	2.17	19.64	1.52
Longli Country of Douyun	1.45	48.40	0.00	0.75	23.82	0.00	0.76	16.74	0.00
Longli Country of Douyun	1.37	43.55	0.00	0.72	21.83	0.00	1.68	34.37	0.00
Longli Country of Douyun	1.04	38.03	0.00	0.43	12.13	0.00	0.60	0.00	0.00
Longli Country of Douyun	0.95	37.86	0.00	0.00	0.00	0.00	0.74	0.00	0.00
Longli Country of Douyun	1.05	37.64	0.00	0.00	12.33	0.00	0.75	0.00	0.00
Longli Country of Douyun	0.72	32.02	0.00	0.00	15.09	0.00	1.47	29.93	0.00
Longli Country of Douyun	0.90	32.38	0.00	0.00	0.00	0.00	1.36	27.65	0.00
Longli Country of Douyun	1.13	37.90	0.00	0.29	13.73	0.00	0.57	0.00	0.00
Longli Country of Douyun	0.98	42.39	0.00	1.05	12.00	0.00	1.56	15.94	0.00
Longli Country of Douyun	0.71	28.97	0.00	0.51	0.00	0.00	1.20	15.75	0.00
Longli Country of Douyun	1.04	43.28	0.00	0.54	22.85	0.61	2.27	44.30	2.80
Longli Country of Douyun	1.23	38.11	0.00	0.59	21.79	0.00	1.56	20.64	1.07
Longli Country of Douyun	1.11	46.99	0.00	0.64	0.00	0.00	0.71	0.00	0.00
Taijiang Country of Qiannan	0.88	21.72	0.00	0.00	10.59	0.00	0.70	0.00	0.00
Taijiang Country of Qiannan	0.69	34.07	0.00	0.36	0.00	0.00	1.26	22.72	0.00
Taijiang Country of Qiannan	0.61	31.41	0.00	0.29	0.00	0.00	1.43	22.80	0.00
Taijiang Country of Qiannan	0.75	34.68	0.91	0.46	0.00	0.00	2.03	40.26	1.04
Taijiang Country of Qiannan	0.45	33.87	0.00	0.32	10.63	0.00	0.83	37.37	0.00
Taijiang Country of Qiannan	0.51	40.08	0.00	0.36	14.77	0.00	1.27	21.86	0.00
Taijiang Country of Qiannan	0.00	34.20	0.00	0.49	15.74	0.00	1.27	28.20	0.00
Taijiang Country of Qiannan	1.23	56.31	0.00	1.32	20.46	0.00	1.30	15.18	0.00
Taijiang Country of Qiannan	0.81	38.97	0.00	0.31	12.87	0.00	1.55	21.50	0.00

	Taijiang Country of Qiannan	0.65	36.81	0.00	0.39	14.41	0.00	1.23	16.19	0.00
Sichuan	Dajing Town of Xichang	0.93	42.28	1.45	0.35	13.05	0.00	1.03	24.12	1.65
	Dajing Town of Xichang	0.51	35.14	1.50	0.00	13.17	0.62	0.61	0.00	0.00
	Dajing Town of Xichang	1.15	46.51	0.71	0.47	17.44	0.00	1.27	12.08	0.00
	Dajing Town of Xichang	0.80	40.49	0.00	0.40	0.00	0.00	0.43	0.00	0.00
	Dajing Town of Xichang	1.13	35.19	1.54	0.52	22.21	0.00	0.63	0.00	0.00
	Dajing Town of Xichang	1.37	42.18	1.31	0.44	14.85	0.00	0.79	0.00	0.00
	Dajing Town of Xichang	0.96	24.87	0.00	1.09	22.43	0.00	1.37	25.38	1.40
	Dajing Town of Xichang	0.00	0.00	0.00	0.00	0.00	0.00	0.00	0.00	0.00
	Zhaojue Country of Xichang	1.65	56.95	1.56	0.39	21.83	0.00	1.33	15.35	3.45
	Zhaojue Country of Xichang	0.49	43.52	0.89	0.00	11.86	0.00	1.17	28.09	0.88
	Zhaojue Country of Xichang	1.22	45.47	1.37	0.36	21.10	0.77	0.81	11.62	2.27
	Zhaojue Country of Xichang	1.47	51.18	2.79	0.40	14.02	0.00	1.66	15.93	1.90
	Zhaojue Country of Xichang	0.74	45.98	0.70	0.00	21.29	0.71	1.13	11.12	0.73
	Zhaojue Country of Xichang	1.27	56.01	0.00	0.00	15.48	0.00	1.36	18.14	0.97
	Zhaojue Country of Xichang	1.43	43.75	1.51	0.37	14.05	0.00	1.42	15.05	2.64
	Zhaojue Country of Xichang	0.68	49.54	1.22	0.00	21.20	0.00	1.09	18.05	1.10
	Zhaojue Country of Xichang	1.59	50.22	3.99	0.52	12.36	0.00	1.36	11.44	1.49
	Zhaojue Country of Xichang	1.37	52.60	2.24	0.64	23.06	0.00	1.40	0.00	0.84
	Xide Country of Liangshan	0.58	28.83	1.72	0.00	10.64	0.00	1.63	13.43	1.25
	Xide Country of Liangshan	0.92	32.05	1.62	0.64	19.40	0.00	2.84	13.44	0.89
	Xide Country of Liangshan	0.99	46.47	1.48	0.00	18.00	0.00	1.60	16.25	0.97
	Xide Country of Liangshan	0.30	42.61	0.90	0.00	20.53	0.00	1.15	31.73	51.80
	Xide Country of Liangshan	1.06	29.90	2.00	0.49	0.00	0.00	10.20	0.00	0.00
	Xide Country of Liangshan	0.43	40.08	2.24	0.00	15.94	0.00	0.68	26.24	16.44
	Xide Country of Liangshan	1.06	30.69	2.16	0.31	16.52	0.00	1.55	0.00	0.78
	Xide Country of Liangshan	0.38	26.30	1.33	0.00	0.00	0.00	1.53	21.09	5.41

Xide Country of Liangshan	0.35	28.82	1.58	0.00	0.00	0.00	3.61	18.54	0.00
Xide Country of Liangshan	0.88	39.87	3.12	0.00	14.42	0.69	1.41	16.99	1.13
Mianning Country of Liangshan	0.53	43.99	0.65	0.41	0.00	0.00	1.85	14.95	0.89
Mianning Country of Liangshan	0.34	47.71	0.77	0.29	11.50	0.74	0.95	0.00	0.00
Mianning Country of Liangshan	0.00	33.53	0.00	0.40	0.00	0.00	0.71	0.00	0.00
Mianning Country of Liangshan	0.00	46.67	0.92	0.00	16.01	0.00	0.50	0.00	0.00
Mianning Country of Liangshan	0.31	29.19	1.33	0.00	0.00	0.00	1.02	18.63	0.00
Mianning Country of Liangshan	0.36	37.20	0.88	0.29	0.00	0.88	1.17	16.47	1.97
Mianning Country of Liangshan	0.00	51.12	0.61	0.37	11.99	0.00	1.44	10.92	0.00
Mianning Country of Liangshan	0.51	47.03	0.66	0.00	0.00	0.00	0.48	18.39	0.81
Mianning Country of Liangshan	0.43	52.26	0.61	0.00	17.04	0.00	0.57	11.76	0.64
Mianning Country of Liangshan	0.00	47.06	0.86	0.00	0.00	0.00	0.43	0.00	0.00
Yanbian Country of Panzhihua	1.01	39.90	1.05	0.00	0.00	0.00	0.91	18.36	1.63
Yanbian Country of Panzhihua	1.46	53.44	0.81	0.54	27.10	0.00	1.19	30.82	2.22
Yanbian Country of Panzhihua	1.23	47.12	1.96	0.41	17.31	0.00	1.22	30.52	4.11
Yanbian Country of Panzhihua	1.38	54.27	1.51	0.00	10.92	0.00	1.36	27.75	4.03
Yanbian Country of Panzhihua	0.88	40.69	1.06	0.00	0.00	0.00	1.58	36.28	1.25
Yanbian Country of Panzhihua	1.12	44.73	1.98	0.00	14.93	0.66	1.75	22.07	2.22
Yanbian Country of Panzhihua	1.00	42.92	1.34	0.33	0.00	0.00	1.11	23.24	2.26
Yanbian Country of Panzhihua	0.93	43.94	1.25	0.47	12.28	0.61	1.24	35.46	2.81
Yanbian Country of Panzhihua	1.14	51.36	2.50	0.58	0.00	0.77	1.66	48.81	4.83
Yanbian Country of Panzhihua	1.66	55.65	1.86	1.37	39.91	3.06	0.57	17.63	0.69
Hanyuan Country of Ya'an	0.83	15.76	1.60	0.75	0.00	0.63	3.05	16.83	1.60
Hanyuan Country of Ya'an	0.67	10.53	1.03	0.55	0.00	0.62	1.85	0.00	0.90
Hanyuan Country of Ya'an	0.00	0.00	0.00	0.78	0.00	0.00	1.15	0.00	0.00
Hanyuan Country of Ya'an	0.00	0.00	0.88	0.44	0.00	0.00	0.96	0.00	0.00
Hanyuan Country of Ya'an	0.00	0.00	0.72	0.80	0.00	0.00	1.14	0.00	0.00

	Hanyuan Country of Ya'an	0.67	0.00	0.98	0.78	0.00	0.73	0.82	0.00	0.00
	Hanyuan Country of Ya'an	0.00	0.00	0.97	0.41	0.00	0.00	1.24	0.00	0.00
	Hanyuan Country of Ya'an	0.52	11.84	0.66	0.55	0.00	0.00	1.73	0.00	0.95
	Hanyuan Country of Ya'an	0.43	0.00	0.62	0.47	0.00	0.00	0.93	0.00	0.00
	Hanyuan Country of Ya'an	0.40	0.00	1.40	0.69	0.00	0.00	0.60	0.00	0.00
Guangxi	Longlin Country of Baise	0.45	36.29	0.00	0.00	0.00	0.00	0.00	15.69	0.00
	Longlin Country of Baise	0.40	31.88	0.76	0.00	11.38	0.00	0.29	20.46	0.74
	Longlin Country of Baise	0.00	29.26	0.00	0.00	0.00	0.00	0.00	12.00	0.00
	Longlin Country of Baise	0.30	30.86	0.00	0.00	0.00	0.00	0.00	20.04	0.00
	Longlin Country of Baise	0.41	36.35	0.00	0.00	0.00	0.00	0.00	55.38	0.00
	Longlin Country of Baise	0.00	20.24	0.00	0.00	0.00	0.00	0.66	27.54	0.70
	Longlin Country of Baise	0.00	29.97	0.00	0.00	0.00	0.00	0.00	0.00	0.00
	Longlin Country of Baise	0.00	29.86	0.00	0.00	13.20	0.00	0.00	16.84	0.60
	Longlin Country of Baise	0.00	31.63	0.00	0.00	11.31	0.00	0.59	32.90	1.10
	Longlin Country of Baise	0.00	25.68	0.00	0.00	0.00	0.00	0.52	20.72	0.00
	Napo Country of Baise	0.00	0.00	0.86	0.00	0.00	0.00	0.00	0.00	0.00
	Napo Country of Baise	0.00	0.00	0.76	0.00	0.00	0.00	0.00	0.00	0.00
	Napo Country of Baise	0.00	0.00	0.74	0.00	0.00	0.00	0.00	0.00	0.00
	Napo Country of Baise	0.00	0.00	0.99	0.00	0.00	0.00	0.00	0.00	0.00
	Napo Country of Baise	0.00	0.00	0.98	0.00	0.00	0.00	0.00	0.00	0.00
	Napo Country of Baise	0.00	0.00	0.78	0.00	0.00	0.00	0.33	0.00	0.91
	Napo Country of Baise	0.31	0.00	0.86	0.00	0.00	0.00	0.00	0.00	1.05
	Napo Country of Baise	0.31	0.00	0.78	0.00	0.00	0.00	0.33	0.00	0.98
	Napo Country of Baise	0.34	0.00	0.00	0.00	0.00	0.00	0.00	0.00	0.97
	Napo Country of Baise	0.37	0.00	0.79	0.00	0.00	0.00	0.00	0.00	0.80
Hunan	Bucheng Country of Shaoyang	1.35	56.24	0.70	0.00	0.00	0.93	0.69	0.00	2.02
	Bucheng Country of Shaoyang	0.90	46.01	0.00	0.38	0.00	0.00	0.82	0.00	0.00

Bucheng Country of Shaoyang	0.73	46.01	1.19	0.29	19.74	0.00	0.87	22.31	0.00
Bucheng Country of Shaoyang	0.45	33.79	1.10	0.00	0.00	0.00	1.50	0.00	1.39
Bucheng Country of Shaoyang	0.77	44.22	0.00	0.00	11.59	0.00	0.76	15.32	0.60
Bucheng Country of Shaoyang	0.93	42.67	0.84	0.00	13.29	0.00	0.80	0.00	0.00
Bucheng Country of Shaoyang	0.74	36.54	0.69	0.00	15.29	0.00	0.82	12.38	1.35
Bucheng Country of Shaoyang	0.76	41.38	0.00	0.00	0.00	0.00	1.09	21.26	2.47
Bucheng Country of Shaoyang	0.57	26.17	0.00	0.00	11.69	0.00	1.15	10.65	0.71
Bucheng Country of Shaoyang	0.62	42.23	1.05	0.00	20.04	0.00	0.65	0.00	0.62
